# Posttranscriptional Regulation of miRNAs Harboring Conserved Terminal Loops

**DOI:** 10.1016/j.molcel.2008.10.013

**Published:** 2008-11-07

**Authors:** Gracjan Michlewski, Sonia Guil, Colin A. Semple, Javier F. Cáceres

**Affiliations:** 1 Medical Research Council Human Genetics Unit, Institute of Genetics and Molecular Medicine, Western General Hospital, Edinburgh EH4 2XU, UK; 2 Cancer Epigenetics and Biology Program (PEBC), Catalan Institute of Oncology (ICO-IDIBELL), 08907 L'Hospitalet (Barcelona), Catalonia, Spain

**Keywords:** RNA, PROTEINS

## Abstract

We recently found that hnRNP A1, a protein implicated in many aspects of RNA processing, acts as an auxiliary factor for the Drosha-mediated processing of a microRNA precursor, pri-miR-18a. Here, we provide the mechanism by which hnRNP A1 regulates this event. We show that hnRNP A1 binds to the loop of pri-miR-18a and induces a relaxation at the stem, creating a more favorable cleavage site for Drosha. We found that approximately 14% of all pri-miRNAs have highly conserved loops, which we predict act as landing pads for *trans*-acting factors influencing miRNA processing. In agreement, we show that 2′O-methyl oligonucleotides targeting conserved loops (LooptomiRs) abolish miRNA processing in vitro. Furthermore, we present evidence to support an essential role of conserved loops for pri-miRNA processing. Altogether, these data suggest the existence of auxiliary factors for the processing of specific miRNAs, revealing an additional level of complexity for the regulation of miRNA biogenesis.

## Introduction

MicroRNAs (miRNAs) are small noncoding RNAs that negatively regulate gene expression of complementary mRNAs (Ambros, 2004; Bartel, 2004; He and Hannon, 2004). They have diverse and unique expression patterns and have been implicated in a number of biological processes (for recent reviews see (Bushati and Cohen, 2007; Chang and Mendell, 2007). Many miRNAs target mRNAs that are involved in proliferation, differentiation, and apoptosis, and accordingly impaired microRNA processing enhances cellular transformation and tumorigenesis (Kumar et al., 2007). The biogenesis of miRNAs involves the cropping and release of hairpin-shaped precursors (pre-miRNAs) in the cell nucleus by the microprocessor complex, comprising the RNase III type enzyme Drosha and its partner DGCR8 (Gregory et al., 2004; Han et al., 2004; Zeng et al., 2005). Subsequent events include the export of pre-miRNAs from the nucleus (Yi et al., 2003; Lund et al., 2004) and further processing in the cytoplasm by the type III ribonuclease Dicer into mature miRNAs (Bernstein et al., 2001; Hutvagner et al., 2001).

It has been shown that in many physiological and pathological conditions, individual miRNAs are subjected to posttranscriptional regulation, both at the level of Drosha and/or Dicer processing (Obernosterer et al., 2006; Thomson et al., 2006). We have recently shown that hnRNP A1, a protein implicated in many aspects of RNA processing, specifically binds to a miRNA cluster containing pri-miR-18a and promotes production of miR-18a above other members of the cluster (Guil and Caceres, 2007). This miRNA is expressed as a cluster of intronic RNAs, the miR-17∼18a∼19a∼20a∼19b-1∼92 microRNA polycistron, and overexpression of this cluster accelerates c-*myc*-induced tumor development in a mouse B cell lymphoma model (He et al., 2005). Thus, the general RNA-binding hnRNP A1 protein acts as an auxiliary factor for the processing of a miRNA precursor, pre-miR-18a, at the level of Drosha processing (Guil and Caceres, 2007).

Here, we have elucidated the mechanism by which hnRNP A1 facilitates miR-18a production. We show that hnRNP A1 binds to the loop of this pri-miRNA and induces a relaxation at the stem, which is important for its processing. Furthermore, we show that 14% of all pri-miRNAs have terminal loops that are well conserved throughout evolution, and we predict that this reflects their requirement for auxiliary factors that bind to this sequence. By using 2′-O-methyl oligonucleotides complementary to conserved terminal loops of the corresponding pri-miRNAs, we could efficiently block their processing. Accordingly, we demonstrate that mutations in the terminal loop of pri-miR-18a that do not affect the structural architecture of the stem abrogate its efficient processing. Altogether, these data suggest the existence of auxiliary factors that bind to conserved terminal loops and facilitate the processing of specific miRNAs, revealing an additional level of complexity for the regulation of miRNA production.

## Results

### Identification of hnRNP A1 Binding Sites in Pri-miR-18a

**Figure 1:**
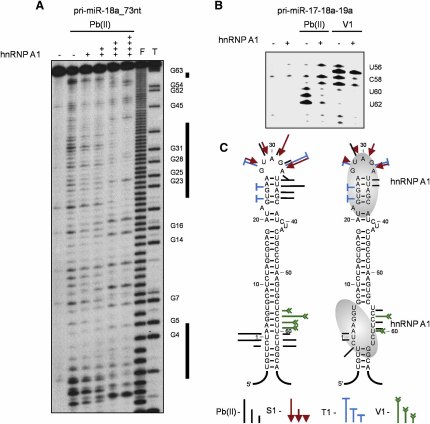
hnRNP A1 Binds to the Terminal and Internal Loops of Pri-miR-18a, Causing Relaxation of the Stem (A) Footprint analysis of the pri-miR-18a_73 nt/hnRNP A1 complex. Cleavage patterns were obtained for 5′ ^32^P-labeled pri-miR-18a transcript (100 × 10^3^ c.p.m.) incubated in the presence of increasing concentrations of recombinant hnRNP A1 protein (+, 50 ng; ++, 100 ng; +++, 150 ng; ++++, 200 ng), treated with Pb (II)-lead ions (0.5 mM). F and T identify nucleotide residues subjected to partial digest with formamide (every nucleotide) or ribonuclease T1 (G-specific cleavage), respectively. Thick lines on the right-hand side indicate rows of nucleotides protected by hnRNP A1. Positions of selected residues are indicated. (B) Cleavage pattern obtained for the unlabeled pri-miR-17-18a-19 RNA cluster incubated in the presence of hnRNP A1 protein (+ 50ng), treated with Pb (II)-lead ions (0.5 mM) and ribonuclease V1 (0.5 u/ml), detected by RT with a pri-miR-18a-specific ^32^P-labeled oligonucleotide. (C) Proposed structure of free and hnRNP A1-bound pri-miR-18a. The sites and intensities of cleavages generated by structure probes (presented below), located at the places of hnRNP A1 binding are shown. Nucleotides are numbered from the 5′ site of Drosha cleavage.

Our previous data showed that hnRNP A1 facilitates processing of miR-18a in a context-dependent manner, emphasizing the importance of the pri-miRNA sequences surrounding miR-18a in the requirement for hnRNP A1 (Guil and Caceres, 2007). In order to map the precise binding site/s of hnRNP A1 in pri-miR-18a and to determine whether this binding influences the RNA architecture and/or thermodynamic properties that might facilitate its processing, we analyzed its RNA secondary structure in the presence or absence of recombinant hnRNP A1 protein. Footprint analysis, using Pb(II)-lead ions that cleave single-stranded and relaxed nucleotides, revealed two hnRNP A1 binding regions: a primary one corresponding to the terminal loop of pri-miR-18a and a secondary site that corresponds to the bottom of the stem (Figure 1A). Interestingly, both sites share some similarity with the consensus hnRNP A1 binding site, UAGGGA/U, that was previously identified by SELEX experiments (Burd and Dreyfuss, 1994). These results were confirmed and extended by the use of a variety of structural probes and by footprint analysis of pri-miR-18a performed in the context of a transcript encompassing the minicluster pri-miR-17-18a-19a. This analysis revealed that the binding of hnRNP A1 to the internal loop in the stem of pri-miR-18a not only confers protection to specific nucleotides, but also results in relaxation of residues between U56 and U60 that are involved in strong Watson-Crick pairing in the unbound pri-miR-18a molecule (Figures 1B and 1C and data not shown). The model shown on Figure 1C is based on multiple experiments with pri-miR-18a with different length of flanking sequences that showed highly reproducible patterns (Figure S1 and data not shown). We also confirmed that within the miR-17-18a-19a minicluster, pri-miR-18a displays the highest affinity toward hnRNP A1, as it was the only pri-miRNA capable of efficiently competing the interaction with hnRNP A1 (Figure S2). Thus, the sequence and natural context of pri-miR-18a might constitute a suboptimal recognition site for Drosha/DGCR8 cropping. It is likely that hnRNP A1 bound to the stem structure in pri-miR-18a acts to unwind or rearrange this RNA structure, creating a more favorable cleavage site for Drosha. This is most likely related to the reported unwinding/annealing activities of hnRNP A1 (Kumar and Wilson, 1990; Pontius and Berg, 1990; Munroe and Dong, 1992).

### hnRNP A1 Acts to Remodel the Stem Structure of pri-miR-18a Facilitating Drosha-Mediated Processing

**Figure 2:**
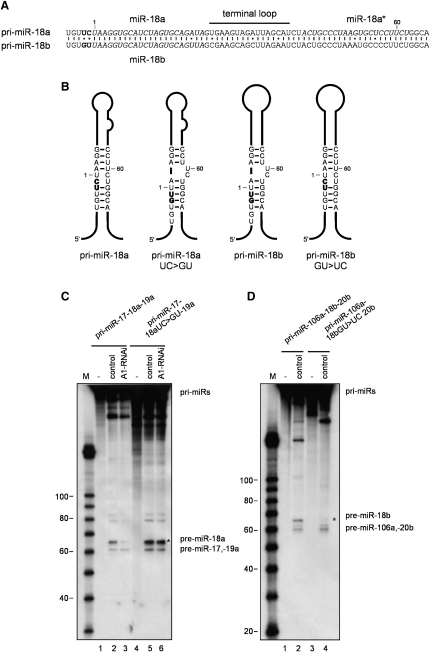
A Fine Structural Alteration in the Pri-miR-18a Stem Allows for hnRNP A1-Independent Drosha Cleavage (A) Sequences of the highly related pri-miR-18a and pri-miR-18b. (B) Schematic of the secondary structures of wild-type and stem mutants of pri-miR-18a and pri-miR-18b. Mutated nucleotides are bolded. (C) In vitro processing of pri-miR-18a and the mutant pri-miR-18a UC > GU substrate. Both radiolabeled primary RNA sequences (50 × 10^3^ c.p.m.) were incubated in the presence of either control HeLa extracts (lanes 2 and 5) or hnRNP A1-depleted extracts (lanes 3 and 6). Lanes 1 and 4 show negative controls with no extract added. Products were analyzed on an 8% polycrylamide gel. The asterisk indicates the pre-miR-18a product. M, RNA size marker. (D) In vitro processing of pri-miR-18b and the mutant pri-miR-18b GU > UC substrate. Both radiolabeled primary RNA sequences (50 × 10^3^ c.p.m.) were incubated in control HeLa extracts (lanes 2 and 4). Lanes 1 and 3 show negative controls with no extract added. Products were analyzed on an 8% polycrylamide gel. The asterisk indicates the pre-miR-18b product. M, RNA size marker.

In order to define potential structural features that make pri-miR-18a processing dependent on hnRNP A1, we took advantage of a highly related pri-miRNA sequence, pri-miR-18b, which is part of the homologous primary cluster miR106a∼18b∼20b located on chromosome X (Tanzer and Stadler, 2004) that was shown to be processed independently of hnRNP A1 (Guil and Caceres, 2007). In the case of pri-miR-18b, we detected an increase in the intensity of Pb(II)-lead ion cleavages between G54 and G63 that mapped to the UC bulge (Figure S3 and Figure 2B). Thus, the cleavage pattern of pri-miR-18b resembles the structural rearrangements seen in the stem of pri-miR-18a in the presence of added recombinant hnRNP A1 protein, that is, the relaxation of residues between U56 and U60 that are involved in strong Watson-Crick pairing (Figure 1A and Figure S3). To establish the significance of this observation, we introduced two nucleotide mutations on pri-miR-18a and pri-miR-18b in order to force structural changes in the stems so that the conformation of the mutant pri-miR-18a resembles the one from pri-miR-18b and vice versa (Figure 2B). Footprint analysis confirmed that the designed mutations altered only locally the conformation of the stem loops without perturbing the rest of the structure (Figure S4). We then performed in vitro processing assays on these new substrates in the context of the pri-miR-17-18a-19a minicluster. As previously reported, miR-18a was the only pri-miRNA whose processing was reduced when incubated in extracts from cells depleted of hnRNP A1, since both the neighboring 17 and 19a pri-miRNAs were insensitive to the reduced levels of hnRNP A1 (Figure 2C, lane 3) (see also (Guil and Caceres, 2007). Upon depletion of hnRNP A1 achieved with an unrelated set of siRNAs, we observed a similar result, and the accumulation of the remaining RNA containing unprocessed pre-miR-18a becomes more evident (Figure S5B, asterisk). Strikingly, introduction of a bulge in the pri-miR-18a stem (UC → GU) made its processing more efficient and completely independent of the presence of hnRNP A1 (Figure 2C). This experiment clearly shows that a pri-miR-18a with a local structural change in its stem resembling pri-miR-18b (or pri-miR-18a upon addition of hnRNP A1 as shown on Figure 1C) is now processed independently of hnRNP A1. By contrast, the mutation in pri-miR-18b that eliminates the UC bulge (GU → UC) did not make this substrate dependent of hnRNP A1 for its processing but rather abrogated its processing in HeLa cell extracts (Figure 2D). From these experiments, we conclude that the requirement for hnRNP A1 as an auxiliary factor in the processing of pri-miR-18a reflects the ability of this factor to bind and alter the local conformation of the stem in the vicinity of Drosha cleavage sites. These results revealed that subtle structural features in pri-miRNAs that are innate or arise from chaperone activities of RNA binding proteins might facilitate their processing by Drosha.

### Conserved Terminal Loops in Pri-miRNAs

**Figure 3:**
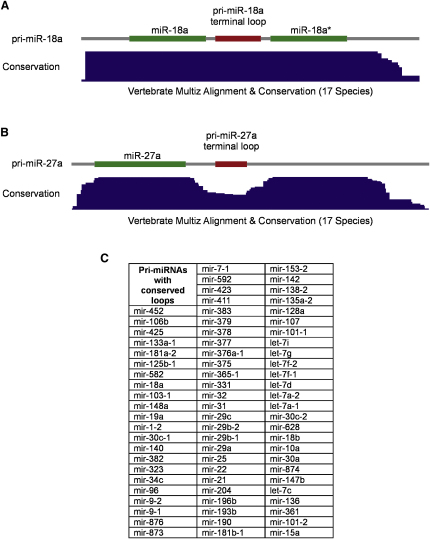
Multiple Sequence Alignments for the Human Genome with 16 Other Vertebrate Genomes Show Very High Level of Conservation in the Terminal Loop Regions of Some Human pri-miRNAs (A) Conservation across pri-miR-18a corresponds to a persistently high phastCons conservation profile (the blue track labeled “Conservation”) across stem and terminal loop region predicted by miRBase and EvoFold. (B) Conservation across pri-miR-27a includes a clear dip in phastCons conservation profile (the blue track labeled “Conservation”) corresponding to the terminal loop region predicted as above. (C) A list of 74 pri-miRNAs with unusually highly conserved terminal loops is shown.

Since the footprint analysis showed that hnRNP A1 has a strong binding site on the pri-miR-18a terminal loop, we next focused on the significance of this interaction. It is assumed that the terminal loops of pri-miRNAs are relatively functionally unimportant (Han et al., 2006). This notion is in agreement with the poor phylogenetic conservation that most pri-miRNAs display along their terminal loop regions, which contrasts with the high level of conservation in the mature miRNA sequences (Berezikov et al., 2005). We have noticed that the terminal loop of pri-miR-18a is atypically well conserved across vertebrate species (Figure 3A, compare with pri-miR-27a, which displays no special conservation in the loop, Figure 3B). Phylogenetic analysis of human pri-miRNAs sequences revealed that ∼14% (74 out of 533) of the miRNAs analyzed had similar high conservation pattern across the whole pri-miRNA sequence (Figure 3C and Figure S6). It is conceivable that the conservation of the terminal loops indicates the need to preserve a platform for the binding of auxiliary factors required for an efficient processing of these pri-miRNAs.

### Oligonucleotides Complementary to Conserved Terminal Loops, LooptomiRs, Block pri-miRNA Processing

**Figure 4:**
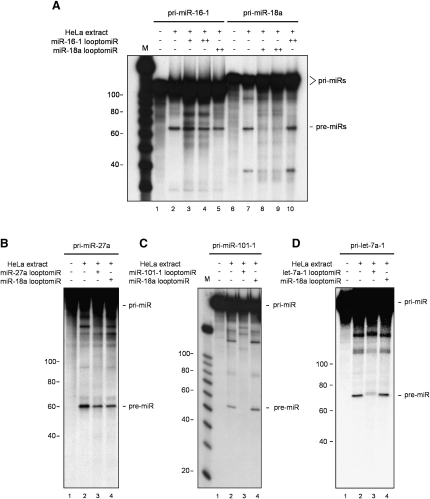
LooptomiRs Designed against Conserved Terminal Loops Block the Processing of a Subset of Pri-miRNAs (A) Primary RNA transcripts corresponding to pri-miR-16-1 (lanes 1–5) and pri-miR-18a (lanes 6–10) (50 × 10^3^ c.p.m.) were processed in HeLa extracts in the presence of specific (lanes 3 and 4 and 8 and 9 for pri-miR-16-1 and pri-miR-18a, respectively) or control (lane 5 for miR-16-1 and lane 10 for miR-18a, respectively) looptomiRs (+, 50 μM; ++, 100 μM). Lanes 1 and 6 show negative controls with no extract added. M, RNA size marker. (B) Processing of pri-miR-27a is not affected by the addition of a specific looptomiR. The RNA substrate (50 × 10^3^ c.p.m.) was processed in vitro in the presence of the corresponding blocking looptomiR (lane 3) or a control looptomiR (lane 4). (C and D) In vitro processing of pri-miRNAs 101-1 and let-7a-1 is sensitive to the presence of specific looptomiRs. Radiolabeled pri-miRNA 101-1 and let-7a-1, (50 × 10^3^ c.p.m.) were processed in HeLa extracts with the addition of a specific looptomiR (lane 3) or with a control looptomiR specific for miR-18a (lane 4). As a control, the reaction was also carried out in the absence of any looptomiR (lanes 2). Lanes 1 represent control reaction without extract. M, RNA size marker.

In this regard and to evaluate the role of conserved terminal loops in pri-miRNA biogenesis, we performed in vitro processing assays in HeLa extracts with the addition of 2′-O-methyl-modified oligonucleotides complementary to sequences in the terminal loops, which we termed LooptomiRs (for Loop Targeting Oligonucleotide anti miRNAs). We found that the Drosha-mediated processing of pri-miR-16-1 and pri-miR-27a, which bear nonconserved terminal loops, was not affected by the addition of complementary looptomiRs (Figures 4A and 4B). This observation was in agreement with a previous study showing that pri-miR-16-1 did not require the terminal loop for its efficient processing (Han et al., 2006). By contrast, looptomiRs designed against a number of conserved terminal loop sequences, including those of pri-miR-18a, pri-miR-101-1, pri-let-7a-1, pri-miR-379, and pri-miR-31, were able to specifically block the processing of their corresponding pri-miRNAs (Figures 4A, 4C, and 4D and Figures S7A and S7B). We believe that this is due to the block exerted by looptomiRs on sequences within the terminal loops that bind auxiliary factors required for the efficient processing of these targeted miRNAs. Furthermore, a looptomiR targeting miR-18a selectively abolishes the processing of pri-miR-18a in the context of the miR-17-18a-19a minicluster (Figure S7C). It is possible that looptomiRs could be used to block access to negative factors that negatively regulated pri-miRNA processing. It is also conceivable that some looptomiRs could potentially change the secondary structure of complementary pri-miRNAs, thus affecting the cleavage by Drosha. However, at least in the case of pri-miR-18a bound to its looptomiR, we did not detect any significant changes in the RNA structure of the region surrounding the Drosha cleavage site (Figure S8).

**Figure 5:**
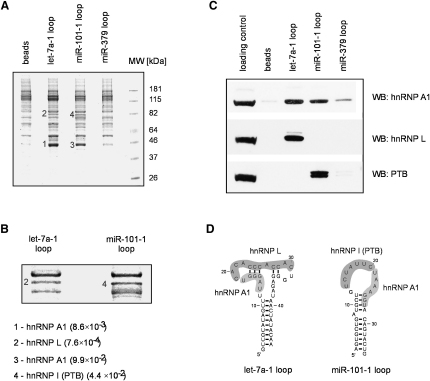
Truncated Stem Loops of Pri-miRNAs with Conserved Terminal Loops Bind Common and Distinct RNA-Binding Proteins (A) RNA chromatography combined with Mass Spectrometry of selected pri-miRNAs harboring conserved loops was performed in HeLa nuclear extracts. Samples resulting from RNA chromatography were resolved on SDS gel and visualized using GelcodeBlue (Pierce). Bands 1 and 3 correspond to hnRNP A1, band 2 corresponds to hnRNP L, and band 4 corresponds to hnRNP I (PTB). (B) A close-up of a region of the gel shown in (A). Expectation values from the mass spectrometry analysis are shown. (C) Western blot analysis of RNA chromatography with truncated stem loops of corresponding pri-miRNAs. (D) Predicted structures of pri-let-7a-1 and pri-miR-101-1 with highlighted putative protein binding sites are shown.

The above results allowed us to conclude that the conserved terminal loop region of pri-miR-18a is essential for its efficient processing, most likely due to the direct binding of the auxiliary factor hnRNP A1. Moreover, the effect of looptomiRs on the processing of pri-miRNAs with conserved loops suggest a more general role of RNA binding proteins as auxiliary factors in the microRNA processing pathway. In order to find out potential additional auxiliary factors regulating processing of pri-miRNAs harboring conserved terminal loops, we combined RNA chromatography with mass spectrometry. Using this approach, we found that hnRNP A1, which was originally defined as an auxiliary factor for miR-18a, also binds to the apical regions of pri-let-7a-1 and pri-miR-101-1 stem loop (Figure 5 and Figure S9). The specificity of this interaction was demonstrated by the lack of hnRNP A1 binding to the loops of mir-16-1, miR-19a, miR-21, and miR-379 (Figure 5C and Figure S9). Notably, the terminal loop of let-7a-1 has a perfect hnRNP A1 consensus-binding site (UAGGGA/U) (Figure 5D). This loop was also found to bind hnRNP L, whereas PTB was found to bind to the pri-miR-101-1 stem loop. Experiments are currently under way to elucidate the role of these factors in the processing of their corresponding pri-miRNAs.

### Pri-miR-18a Complex Formation

In order to have a closer look at the nature of the interaction between pri-miR-18a and hnRNP A1, we performed electrophoretic gel mobility shift (EMSA) experiments. First, we assayed increasing amounts of radiolabeled pri-miR-18a (the same transcript as described in Figure 4) programmed with a constant amount of hnRNP A1 recombinant protein (200 nM). The molar ratio of pri-miR-18a relative to hnRNP A1 ranged from 0.25 to 4, such that RNA binding will saturate the protein. A monomeric complex, migrating in between the substrate and its minor structural conformer, had a binding stoichiometry close to one (Figure S10A). A larger multimeric complex migrating above the minor conformer was formed in the higher RNA:protein molar ratios, indicating that pri-miR-18a is able to bind more than one molecule of hnRNP A1. Importantly, addition of a specific looptomiR targeting pri-miR-18a abrogated the formation of the monomeric miR-18a/hnRNP A1 complex without substantially affecting the multimeric complex (Figure S10B, lanes 5–8). We have shown that addition of looptomiRs specifically blocks in vitro miRNA processing (Figure 4 and Figure S7). Taken together, this would suggest that displacing hnRNP A1 from the terminal loop of pri-miR-18a, without affecting its binding to other regions of this pri-miRNA, is sufficient to block its activity in miRNA processing. Moreover, addition of the looptomiR caused a small shift in the migration of both main pri-miR-18a substrate and its minor conformer. This result points to an efficient binding of the looptomiR to the transcript and also suggests small changes of the global architecture of pri-miR-18a stem-loop structure in the presence of the specific looptomiR, which is in agreement with the structural analysis of such complex (Figure S8).

### Role of Terminal Conserved Loops in miRNA Processing

**Figure 6:**
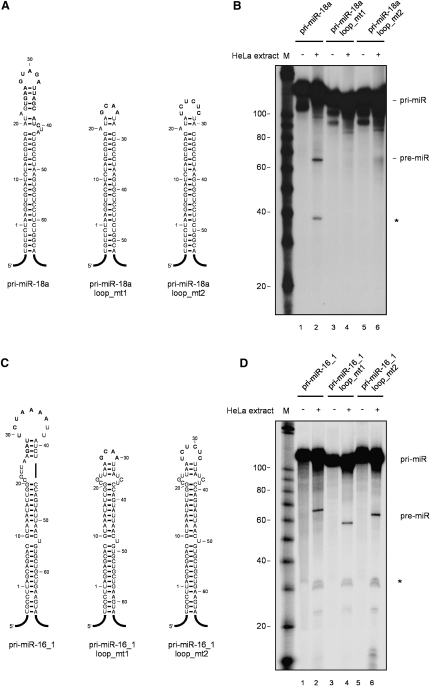
The Conserved Terminal Loop of pri-miR-18a Is Required for Its Efficient Cleavage by Drosha (A) Validated secondary structures of wild-type and terminal loop mutants of pri-miR-18a (pri-miR-18a_loop_mt1 and pri-miR-18a_loop_mt2). Mutated nucleotides are bolded. (B) In vitro processing of wild-type pri-miR-18a and loop mutants. Radiolabeled pri-RNAs (50 × 10^3^ c.p.m.) were incubated in HeLa cell extracts (lanes 2, 4, and 6). Lanes 1, 3, and 5 show negative controls with no extract added. Products were analyzed on an 8% polycrylamide gel. The asterisk indicates the single-stranded RNA regions after Drosha cleavage. M, RNA size marker. (C) Predicted secondary structures of wild-type and terminal loop mutants of pri-miR-16-1. (D) In vitro processing of pri-miR-16-1 and loop mutants pri-miR-16-1_loop_mt1 and pri-miR-16-1_loop_mt2. Radiolabeled pri-RNAs (50 × 10^3^ c.p.m.) were incubated in HeLa cell extracts (lanes 2, 4, and 6). Lanes 1, 3, and 5 show negative controls with no extract added. Products were analyzed on an 8% polycrylamide gel. The asterisk indicates the single-stranded RNA regions after Drosha cleavage. M, RNA size marker.

**Figure 7:**
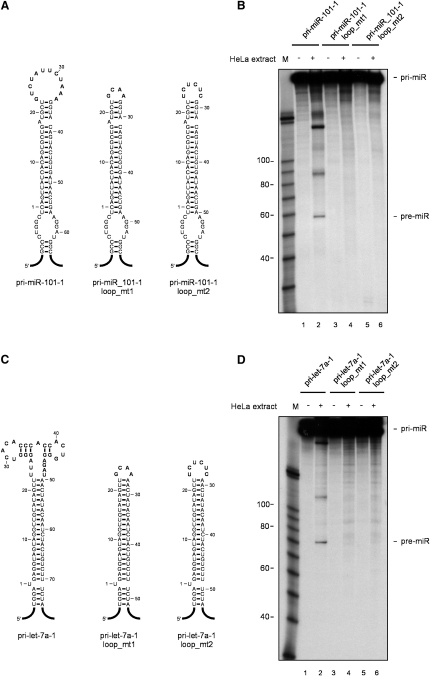
The Conserved Terminal Loops of Pri-miR-101-1 and Pri-let-7a-1 Are Required for Their Efficient Cleavage by Drosha (A) Predicted secondary structures of wild-type and terminal loop mutants of pri-miR-101-1 (pri-miR-101-1_loop_mt1 and pri-miR-101-1_loop_mt2). Mutated nucleotides are bolded. (B) In vitro processing of wild-type pri-miR-101-1 and loop mutants. Radiolabeled pri-RNAs (50 × 10^3^ c.p.m.) were incubated in HeLa cell extracts (lanes 2, 4, and 6). Lanes 1, 3, and 5 show negative controls with no extract added. Products were analyzed on an 8% polycrylamide gel. M, RNA size marker. (C) Predicted secondary structures of wild-type and terminal loop mutants of pri-let-7a-1. (D) In vitro processing of pri-let-7a-1 and loop mutants. Radiolabeled pri-RNAs (50 × 10^3^ c.p.m.) were incubated in HeLa cell extracts (lanes 2, 4, and 6). Lanes 1, 3, and 5 show negative controls with no extract added. Products were analyzed on an 8% polycrylamide gel. M, RNA size marker.

To gain a more clear insight into the role of conserved terminal loops for miRNA processing, we have created pri-miRNAs terminal loop mutants of pri-miR-18a and as a control of pri-miR-16-1 (Figures 6A and 6C). In order to eliminate any potential hnRNP A1 binding site from the pri-miR-18a apical region, we have substituted the region spanning from U24 to C40 with GCAA or UCUCUC loops (miR-loop_mt1 and miR-loop_mt2, respectively). We found that the introduction of mutant sequences in the terminal loop of pri-miR-18a completely abrogated its processing in HeLa extracts, as compared to a wild-type transcript (Figure 6B). This was also true in the context of pri-miR-17-18a-19a minicluster (Figure S11). By contrast, introduction of similar mutant loops to pri-miR-16-1 transcripts didn't influence the rate of pri-miR to pre-miR processing, reinforcing previous findings that pri-miR-16-1 could be processed in the absence of its terminal loop (Figure 6D) (Han et al., 2006). Importantly, we needed to establish whether the mutant loop sequences did not affect the global architecture and thermodynamics of the pri-mir-18a stem. To address this, we performed detailed structure analysis of pri-miR-18a loop mutants, using Pb(II)-lead ions as a probe, which are able to detect potential fine thermodynamic and structural perturbations within the stem-loop region. We found that the pattern of cleavages obtained for wild-type pri-miR-18a and two loop mutants, apart from the terminal loop regions, were identical (Figure S12). This indicates that introduction of different terminal loops to pri-miR-18a does not change the global architecture of its stem region. Finally, we also observed that the introduction of mutant sequences in the conserved terminal loops of pri-miR-101-1 and pri-let-7a-1 completely abrogated their processing in HeLa extracts, confirming the importance of conserved terminal loop regions for efficient miRNA processing (Figures 7B and 7D).

In summary, we have presented here the mechanism by which an auxiliary factor, hnRNP A1, promotes the Drosha-mediated processing of pri-miR-18a. The conservation of terminal loops of a subset of miRNAs together with the inhibition of processing mediated by LooptomiRs strongly suggest the existence of additional auxiliary factors binding to conserved terminal loop sequences and regulating the processing of specific miRNAs.

## Discussion

The expression of mammalian miRNAs can be regulated at the posttranscriptional level by modulating nuclear and cytoplasmic miRNA processing events. For instance, during early mouse development, many pri-miRNAs, including pri-let-7a-1, are present at high levels but are not processed by Drosha, suggesting an important role for posttranscriptional regulation of miRNA processing. Furthermore, an analysis of gene expression in tumor tissues showed widespread discrepancies in the levels of miRNAs and their corresponding primary transcripts (Thomson et al., 2006). In mouse and zebrafish, few miRNAs are expressed in early embryos, and large numbers of miRNAs are induced in temporal and spatial patterns during mid- to late embryonic development (Kloosterman et al., 2006). It has been shown this is not due to transcriptional control. Interestingly, the core enzymes (Drosha and DGCR8) are widely expressed, and no posttranslational regulation of Drosha or Dicer has been reported (Thomson et al., 2006). It is possible that the downregulation of miRNAs during early development could be attributed to a defect in the relative amount and/or activity of auxiliary factors required for microRNA processing. It has also been suggested that cleavage of pre-miRNAs in the cytoplasm by Dicer is regulated in such a way that the presence of the functional mature miRNA is restricted to only a fraction of the tissues where the pre-miRNA is initially expressed (Obernosterer et al., 2006). In most cases, the mechanism by which this fine regulation of miRNA expression is achieved is not yet understood.

Here, we have shown that hnRNP A1 binding to the pri-miR-18a terminal loop and to a region within the stem rearranges the RNA secondary structure, allowing an efficient processing by the Drosha/DGCR8 complex. The question of how widespread the contribution of hnRNP A1 and other auxiliary factors to pri-miRNA processing events is beginning to emerge. We have also found that hnRNP A1 binds with specificity to the conserved loops of pri-miR-101-1 and pri-let-7a-1, and functional validation of the role of hnRNP A1 in the processing of these miRNAs is now under progress (Figure 5 and Figure S9). Thus, data presented here clearly establishes hnRNP A1 as a positive regulator of miR-18a processing (and perhaps of other miRNAs), acting at the level of Drosha processing. Another example of a positive regulator of miRNA processing is illustrated by the effect of TGF-β and BMP signaling in promoting the expression of miR-21, which results in an induction of a contractile phenotype in human vascular smooth muscle cells. In this situation, TGF-β and BMP-specific SMAD signal transducers are recruited to pri-miR-21 in a complex with the RNA helicase p68 and facilitate its Drosha-mediated processing (Davis et al., 2008). Interestingly, several RNA helicases and hnRNP proteins were found associated with the microprocessor complex, but their functions remain to be determined (Gregory et al., 2004). In the case of p68 and p72 DEAD-box helicases, both proteins were shown to be required for pri-miRNA processing in the mouse (Fukuda et al., 2007). The developmentally regulated RNA-binding protein Lin28 protein has been recently shown to be necessary and sufficient to block the Drosha-mediated cleavage of pri-let-7 miRNAs in embryonic cells (Piskounova et al., 2008; Viswanathan et al., 2008). There is, however, a conflicting report indicating that Lin28 could be acting at the level of Dicer processing (Rybak et al., 2008). In any case, irrespective of the precise mechanism, Lin28 can be classified as a negative regulator of pri-miRNA processing. Interestingly, Lin28 has been shown to bind to conserved nucleotides in the terminal loop region of the Let-7d precursor (Newman et al., 2008). Several hnRNP proteins, among them hnRNP A1, were also found associated with Let-7d, but their functional role was not established (Newman et al., 2008).

Thermodynamic properties and molecular architectures of pri-miRNAs that allow cleavage and release of the pre-miRNAs from their primary transcripts are beginning to be recognized (Krol et al., 2004; Han et al., 2006). It was proposed that a large terminal loop is critical for miRNA processing (Zeng et al., 2005); however, more recent data suggested that the terminal loop is unessential, whereas the flanking single-stranded RNA segments are critical to define the cleavage site. This model predicts that the microprocessor component, DGCR8, functions as a molecular anchor that measures the distance from the dsRNA-ssRNA junction and positions the processing center of Drosha ∼11 bp up along the stem (Han et al., 2006). Our findings indicate that the fine structural features of pri-miRNAs (innate or arising from chaperone activities of RNA binding proteins) might be very important in the miRNA biogenesis pathway. A bioinformatic survey revealed that a number of pri-miRNAs are conserved throughout vertebrate evolution not only at the mature miRNA sequence but also at the regions encompassing terminal loops (Figure 3 and Figure S6). Notably, it was recently shown that the processing of pri-miR-375 in zebrafish was effectively blocked by injecting morpholino oligonucleotides targeting the mature miRNA or the miRNA precursor, some of which overlap the terminal loop, which as shown in Figure S6, is highly conserved (Kloosterman et al., 2007). Results presented in this study support the notion that phylogenetic conservation of pri-miRNA terminal loops reflects the requirement for auxiliary factors that bind to these conserved loops and ensure an optimal pri-miRNA processing. Altogether, our findings open up additional avenues toward a deeper understanding of microRNA biogenesis pathways and their contribution to physiological states and pathological conditions.

## Experimental Procedures

### Preparation of Plasmids and DNA Templates

For the structural analysis, RNA substrates were transcribed in vitro from a DNA fragment containing the T7 promoter sequence directly upstream of the pri-miRNA precursor sequence. The DNA fragments were amplified by PCR from human genomic DNA using oligonucleotides, which are listed in the Supplemental Data.

### RNA Probing and Footprint Analysis

In vitro transcription of RNAs subjected to structure probing was carried out as previously described (Sobczak et al., 2003). Transcripts were 5′ end-labeled with T4 polynucleotide kinase (Roche) and [γ-^32^P] ATP (4,500 Ci/mmol; Amersham Pharmacia Biotech). Transcripts were purified by electrophoresis in a 10% denaturing polyacrylamide gel. Labeled and unlabeled RNAs were visualized by autoradiography and StainsAll staining, respectively. Prior to structure probing transcription, reactions were subjected to a denaturation and renaturation procedure in a buffer containing 2 mM MgCl_2_, 80 mM NaCl, and 20 mM Tris–HCl (pH 7.2) by heating the sample at 80°C for 1 min and then slowly cooling to 37°C. Limited RNA digestion was initiated by mixing 5 μl of the RNA sample (100 000 c.p.m. or 100 pmol for labeled and unlabeled RNAs, respectively) with 5 μl of a probe solution containing lead ions-Pb(II), nuclease S1, or ribonucleases T1 or V1 at concentrations specified in the Figure Legends. The reactions were performed at 37°C for 10 min. For the footprint analysis, recombinant hnRNP A1 protein was added in specified concentrations 5 min prior to the reaction. All reactions were carried out with 5′ ^32^P-labeled RNAs and were stopped by adding an equal volume of stop solution (7.5 M urea and 20 mM EDTA with dyes) and sample freezing. Unlabeled RNAs, after incubation with the probes, were subjected to high-salt precipitation procedure. To determine the cleavage sites, the products of the 5′ ^32^P-labeled RNA fragmentation reaction along with the products of alkaline hydrolysis and limited T1 nuclease digestion of the same RNA molecule were separated on 10% polyacrylamide gels containing 7.5 M urea, 90 mM Tris-borate buffer, and 2 mM EDTA. The alkaline hydrolysis ladder was generated by incubation of labeled RNA in formamide containing 0.5 mM MgCl_2_ at 100°C for 10 min. Partial T1 ribonuclease digestion of RNAs was performed under semidenaturing conditions (10 mM sodium citrate (pH 5.0); 3.5 M urea) with 0.2 U/μl of the enzyme and incubation at 55°C for 15 min. Electrophoresis was performed at 1500 V and was followed by autoradiography at −80°C with an intensifying screen or exposed to the PhosphorImager screen. To determine the cleavages sites obtained for unlabeled RNAs, a reverse transcription-based reaction was used as previously described (Michlewski and Krzyzosiak, 2004). Briefly, 100 ng of RNA was reverse transcribed with the use of 2 pmol 5′ ^32^P-labeled pri-miR-18a rev primer and SuperScript II reverse transcriptase. The products of the reverse transcription were separated as described above, along with the sequence ladder generated from the pri-17-18a-19a using Promega fentomol sequencing kit.

### Pri-miRNA Substrates and In Vitro Processing Assays

RNA substrates for in vitro processing assays were prepared from the DNA templates by standard in vitro transcription with T7 RNA polymerase in the presence of [α-^32^P]GTP. Preparation of total or hnRNP A1-depleted HeLa extracts was described previously (Guil and Caceres, 2007). Assays were done in 30 μl reaction mixtures containing 50% (v/v) total or depleted HeLa extract, 0.5 mM ATP, 20 mM creatine phosphate, 3.2 mM MgCl_2_, and 20,000 c.p.m. (∼10 fmol) of each pri-miRNA. Reactions were incubated at 30°C for 30 min, then subjected to phenol-chloroform extraction, precipitation, and 8% (w/v) denaturing gel electrophoresis. For terminal loop blocking with looptomiRs, specific or control 2′-O-methyl oligonucleotides were purchased from Sigma-Aldrich and added to the pri-miRNA at a final concentration of 50 μM 5 min prior to the incubation with extract to allow annealing.

The looptomiRs sequences used were as follows: anti miR-16-1 loop (5′-oToAoAoToToToToAoGoAoAoToCoToToA-3′), anti miR-18a loop (5′-oAoToGoCoToAoAoToCoToAoCoToToCoA-3′), anti miR-27a loop (5-oGoAoCoToToGoGoToGoToGoGoAoCoCoC-3′), anti let-7a-1 loop (5′-oGoGoToGoGoGoToGoToGoAoCoCoCoToA-3′), anti pri-miR-101-1 (5′-oCoCoToToToAoGoAoAoToAoGoAoCoAoG-3′), anti pri-miR-31 (5′- oGoGoToToCoCoCoAoGoToToCoAoAoCoA-3′), anti pri-miR-379 (5′-oGoToCoAoGoAoAoAoToCoAoToAoAoCoG-3′). An Ambion's Decade RNA Size Marker was used.

### Purification of Recombinant Protein

Recombinant hnRNP A1 protein was obtained as previously described (Cazalla et al., 2005). In brief, T7 tagged hnRNP A1 protein was expressed in the 293T cells transfected with pCG-hnRNP A1 vector (Caceres et al., 1997). Forty-eight hours after transfection, cells were scrapped and sonicated in lysis buffer. Recombinant hnRNP A1 protein was purified using anti-T7 affinity chromatography.

### Phylogenetic Sequence Analysis

Predicted structures and genomic mappings for all 533 known human pri-miRNAs were obtained from miRBase release 10. Of these, only 5 pri-miRNAs were unmapped in the current human genome assembly (hg18/NCBI36): miR-672, miR-941-4, miR-674, miR-872, and miR-871. Coordinates for five subregions (prestem, stem1, loop, stem2, and poststem) within each miRNA were based upon miRBase structures. These coordinates were then reconciled with gapped sites in miRBase structures to derive genomic coordinates for all subregions. Multiple sequence alignments for the human genome (hg18/NCBI36 assembly) with 16 other vertebrate genomes (chimp [November 2003, panTro1], macaque [January 2006, rheMac2], mouse [February 2006, mm8], rat [November 2004, rn4], rabbit [May 2005, oryCun1], dog [May 2005, canFam2], cow [March 2005, bosTau2], armadillo [May 2005, dasNov1], elephant [May 2005, loxAfr1], tenrec [July 2005, echTel1], opossum [January 2006, monDom4], chicken [Feb 2004, galGal2], frog [October 2004, xenTro1], zebrafish [May 2005, danRer3], tetraodon [February 2004, tetNig1], and fugu [August 2002, fr1]) were obtained from the UCSC Genome Bioinformatics group (http://genome.ucsc.edu/). Overall conservation scores per bp based upon these alignments (calculated using phastCons) (Siepel et al., 2005) were obtained from the same source. UCSC EvoFold (Pedersen et al., 2006) alignments and secondary structure predictions were used to examine conservation at finer scales. Comparisons of average conservation scores for different miRNA subregions were based upon the mean and 95% confidence intervals for phastCons scores (Piriyapongsa and Jordan, 2007). A total of 526 known miRNAs could be mapped to the current human genome assembly and successfully related to phastCons conservation data. Of this total, 96 pri-miRNAs could not be processed by the current protocol as conservation was so low across all subregions that comparisons were meaningless. The remaining 430 pri-miRNAs were all processed successfully, and 298 (69%) of these showed a significant difference in conservation between stem and loop subregions. Most of these pri-miRNAs show a mean conservation ratio of loop/stem < 1, including pri-mir-10b, indicating a relatively poorly conserved loop. Of the 430 processed pri-miRNAs, 132 (31%) did not show a significant difference between stem and loop regions, but many of these also show loop/stem mean conservation ratios < 1 and/or show low conservation (mean phastCons score < 0.5) across the entire pri-miRNA sequence. Only 74 (17%) miRNAs, including hsa-miR-18a, were found to have a loop/stem ratio close to 1 (within 0.001 from 1) and mean phastCons scores > 0.5 for the entire sequences. This set of 74 sequences therefore constitutes the best candidates for miRNAs with similar processing requirements to hsa-miR-18a (Figure 2C). The table in Figure 3C shows the candidates ranked by mean conservation ratio and suggests that hsa-mir-18a (ranked 8th out of 74) is among the best-predicted candidates.

A detailed section covering phylogenetic sequence analysis methods used in this paper is presented in the Supplemental Data.

## Supplemental Data

Document S1. 12 Figures and Supplemental Experimental Procedures
