# Supplementary material for: Posttranscriptional Regulation of miRNAs Harboring Conserved Terminal Loops
Source: Mol Cell. 2008 Nov 7;32(3-2):383–93. doi: 10.1016/j.molcel.2008.10.013 (PMC2631628; doi:10.1016/j.molcel.2008.10.013)
Supplement: Document S1. 12 Figures and Supplemental Experimental Procedures [file mmc1.pdf]

Molecular Cell, Volume *32*

Supplemental Data

Posttranscriptional Regulation of miRNAs

Harboring Conserved Terminal Loops

Gracjan Michlewski, Sonia Guil, Colin A. Semple, and Javier F. Cáceres

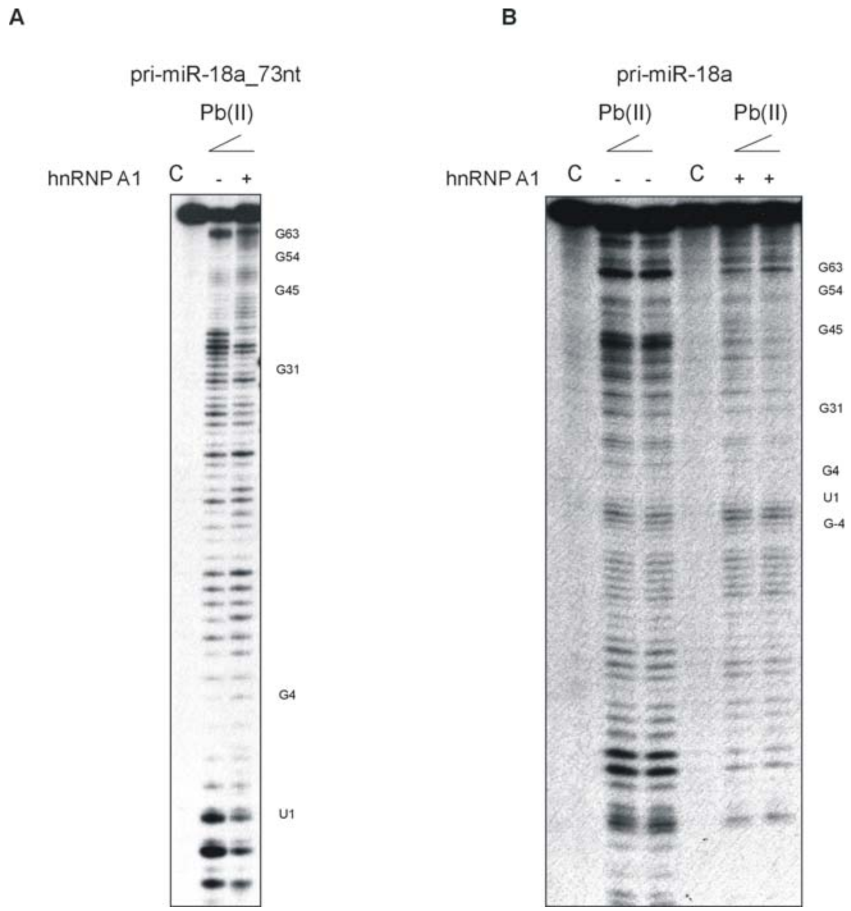

Figure S1. hnRNP A1 primarily binds to the terminal loop of pri-miR-18a transcripts with different length of neighboring sequences (A) Footprint analysis of the pri-miR-18a_73nt/hnRNP A1 complex as shown on Figure 1A. Cleavage patterns were obtained for 5' ^{32}P-labeled pri-miR-18a_73nt transcript (100x10^3 c.p.m.) incubated in the presence or absence of recombinant hnRNP A1 (100ng), treated with Pb (II) – lead ions (0.5mM). Positions of selected residues are indicated (B) Footprint analysis of the pri-miR-18a/hnRNP A1 complex. Cleavage pattern obtained for 5' ^{32}P-labeled pri-miR-18a (100x10^3 c.p.m.) incubated in the presence or absence of recombinant hnRNP A1 (100ng), treated with Pb (II) – lead ions (0.25mM and 0.5mM). Analysis and data interpretation was performed as described in the legend to Figure 1.

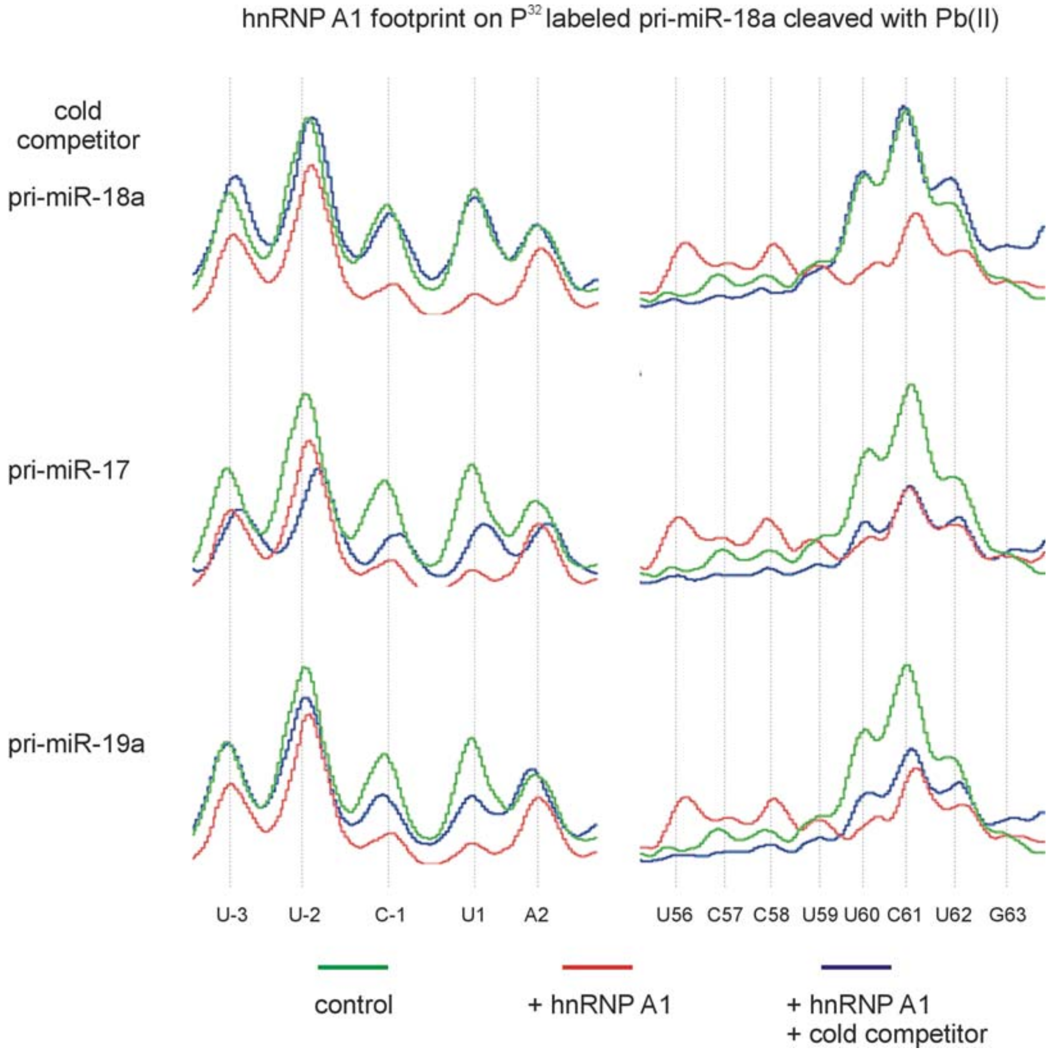

Figure S2. Pri-miR-18a has a high affinity towards hnRNP A1 binding. PhosphorImager peaks represent Pb (II) – lead ions (0.5mM) cleavage patterns of a 5' ^{32}P-labeled pri-miR-18a transcript (100x10^3 c.p.m.) in the absence (green lines) or presence of recombinant hnRNP A1 (100ng) (red lines). When indicated reactions were supplemented with addition of an excess of cold pri-miRNAs competitor (500ng) (blue lines). Nucleotides are numbered as described in the legend to Figure 1.

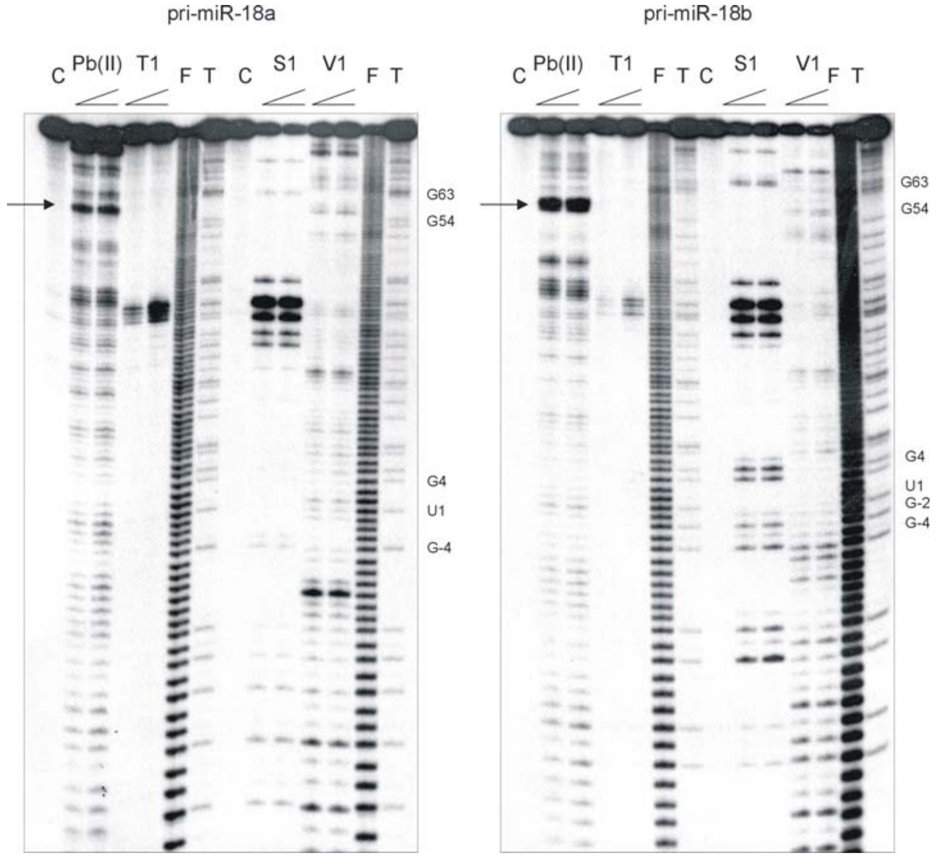

Figure S3. Structure analysis of pri-miR-18a and pri-miR-18b transcripts reveals a fine difference in the stem architecture. Cleavage patterns obtained for the 5' ^{32}P-labeled pri-miR-18a (left panel) and pri-miR-18b (right panel) transcripts (100x10^3 c.p.m.) treated with: Pb (II) – lead ions at increasing concentrations (0.5, 1 mM), T1, ribonuclease T1 (1, 1.5 units/µl), S1, nuclease S1 (1.25, 2.5 units/µl; 1 mM $ZnCl_2$ was present in each reaction mixture) and V1, ribonuclease V1 (0.037, 0.075 unit/ml). C, incubation control (without probe). F and T identify nucleotide residues subjected to partial digest formamide (every nucleotide) or with ribonuclease T1 (G-specific cleavage), respectively. Electrophoresis was performed in a 10% polyacrylamide gel under denaturing conditions. The positions of selected G residues are shown. The arrows indicate a dinucleotide mismatch in pri-miR-18a and a dinucleotide bulge in pri-miR-18b, respectively.

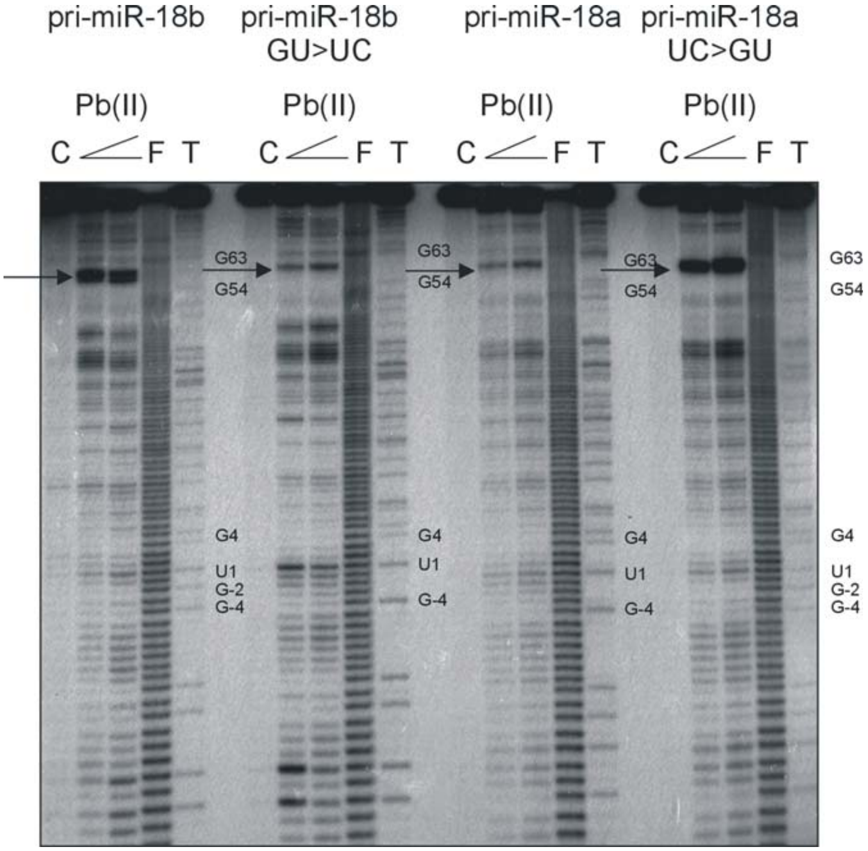

Figure S4. Swapping mutations in the stems of pri-miR-18a and 18b force local changes in the conformation of the stem loops, without perturbing the rest of the structure. Condition of analysis and abbreviations are as described for Figure S3.

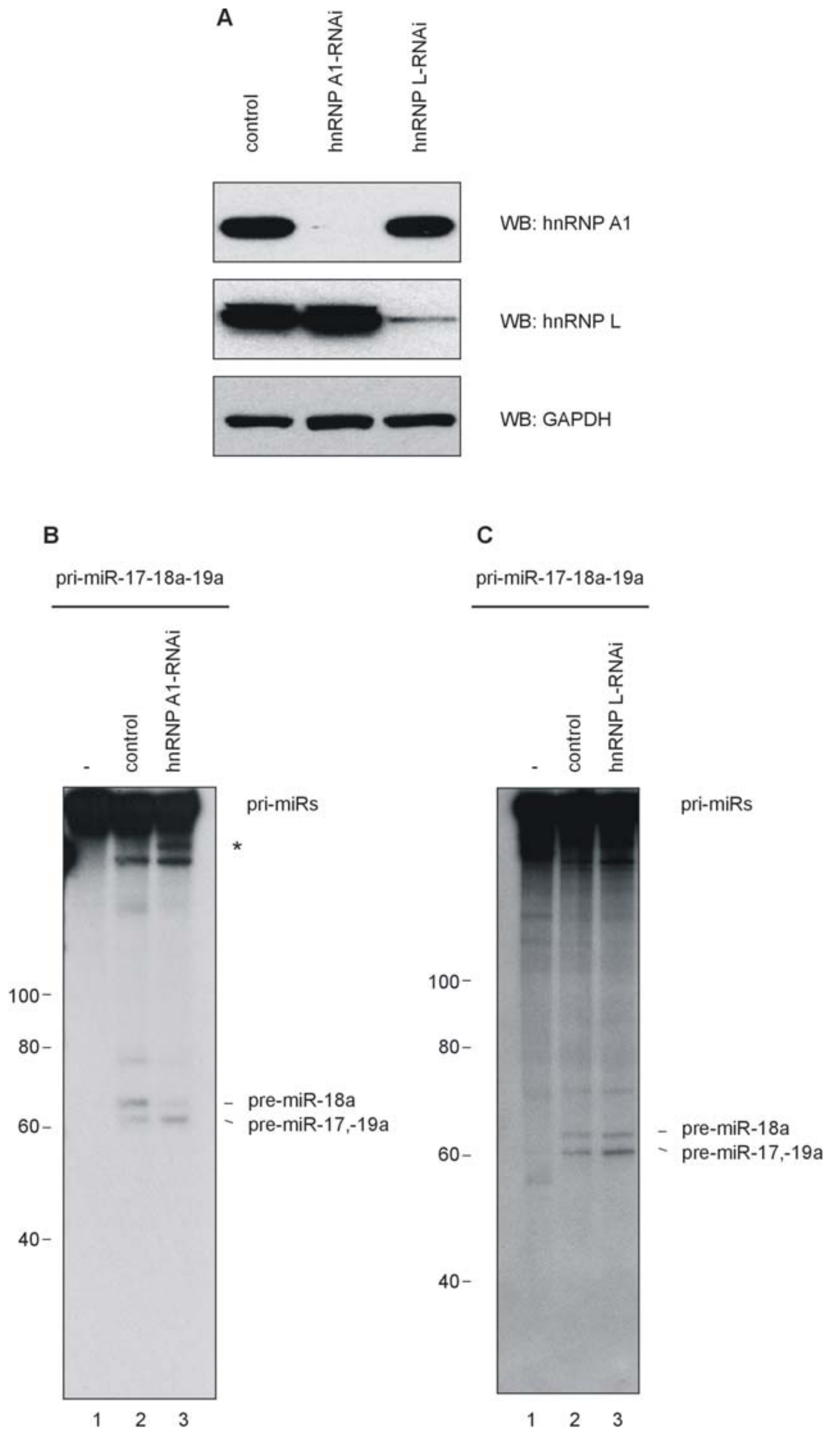

Figure S5. (A) Western blot analysis of HeLa extracts depleted of hnRNP A1 or hnRNP L using specific siRNAs. (B) The processing of pri-miR-18a is dependent on the presence of hnRNP A1. *In vitro* processing of pri-miR-18a in the pri-miR-17-18a-

19a mini cluster is abolished in HeLa cell extracts prepared using different set of siRNAs (hnRNP A1-targeted smart pool of siRNAs from Dharmacon). Primary RNA transcripts were incubated in the presence of either control HeLa extract (lane 2) or hnRNP A1-depleted extract (lane 3). Lane 1 shows negative controls with no extract added. Products were analyzed on an 8% polycrylamide gel. The asterisk indicates the remaining transcript containing uncleaved pre-miR-18a substrate. (C) The processing of pri-miR-18a is no affected by depletion of hnRNP L. Analysis and data interpretation was performed as described for panel B.

miRNA genes showing significant conservation of predicted loop regions. In each case the miRBase identifier is given followed by the chromosomal position (and strand), the miRBase predicted secondary RNA structure and a diagram displaying overall conservation throughout vertebrate evolution. Each diagram indicates the position of the miRNA (red line), predicted loop position (hatched black line), overall PhastCons conservation score (blue graph) and conservation within available vertebrate species.

hsa-mir-452 chrX:150878756-150878840 (-)

```
  u          AAC G        G  A        uuug
gc aagcacuuac   U UUUGCAGA GA ACUGAgac    u
|| ||||||||||   | |||||||| || ||||||||    a
cg uucGUGAAUG   A AAACGUCU CU ugacucug    a
  u          --A G        A  C        uauc
```

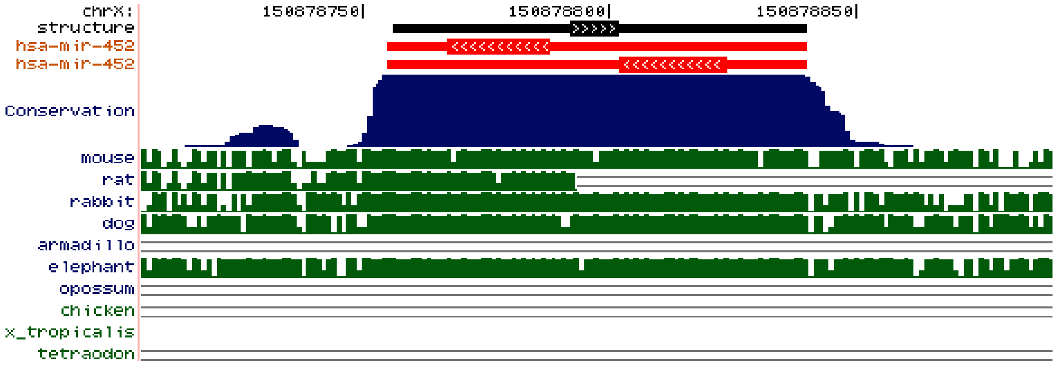

hsa-mir-106b chr7:99529552-99529633 (-)

```
     c     -UA       G       A A    --  uc
ccugc ggggc   AAGUGCU ACAGUGC G Uagu   gg   c
||||| |||||   ||||||| ||||||| | ||||   ||
ggacg ccuCG   UUCAUGG UGUCACG C aucg   cc   u
     a     UCG       G       C -    ug  uc
```

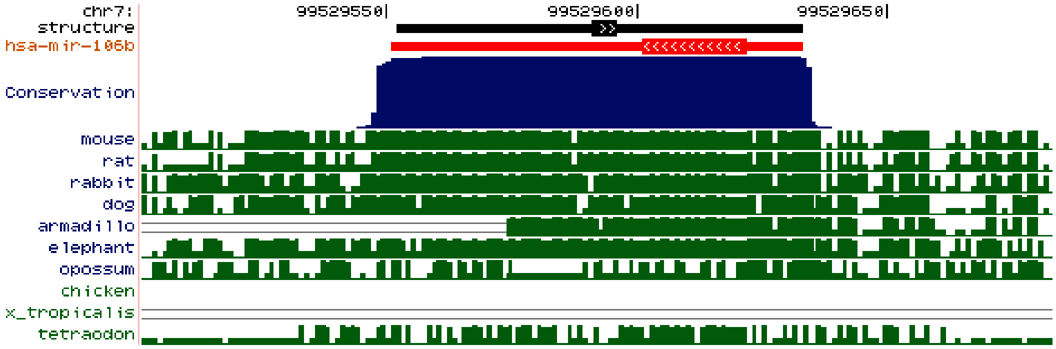

hsa-mir-425 chr3:49032585-49032671 (-)

```
     c  u    AAU       U  C     U  A  -----  g
gaaag gc uugg   GACACGA CA UCCCG UG gu     gg c
||||| || ||||   ||||||| || ||||| || ||     ||
cuuuc cg gaCC   CUGUGCU GU AGGGC Ac cg     cc a
     u  u    CGC       -  A     U  -  aagag  c
```

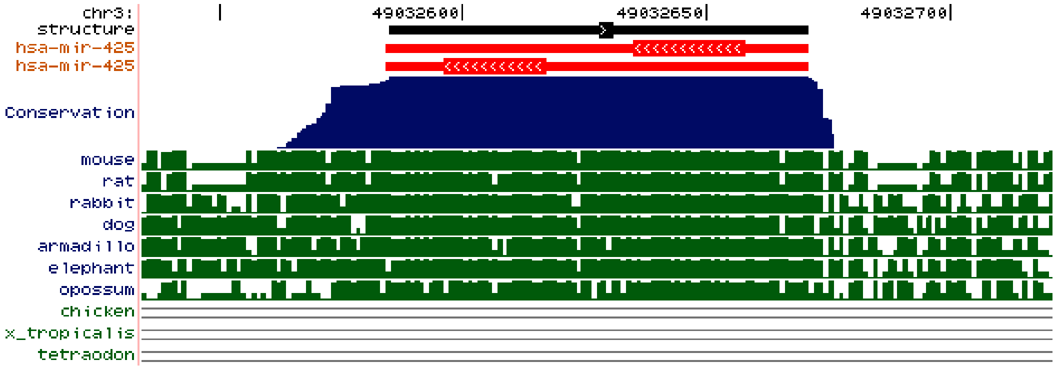

hsa-mir-133a-1   chr18:17659657-17659744 (-)

```
a      uuu    g       aa  u  a        gccuc
 caaugc   gcua agcuggu  aa gg accaaauc     u
 ||||||   |||| |||||||  || || ||||||||
 guuacg   cgau UCGACCA  UU CC UGGUUUag     u
a      uau    G       AC  C  C        guaac
```

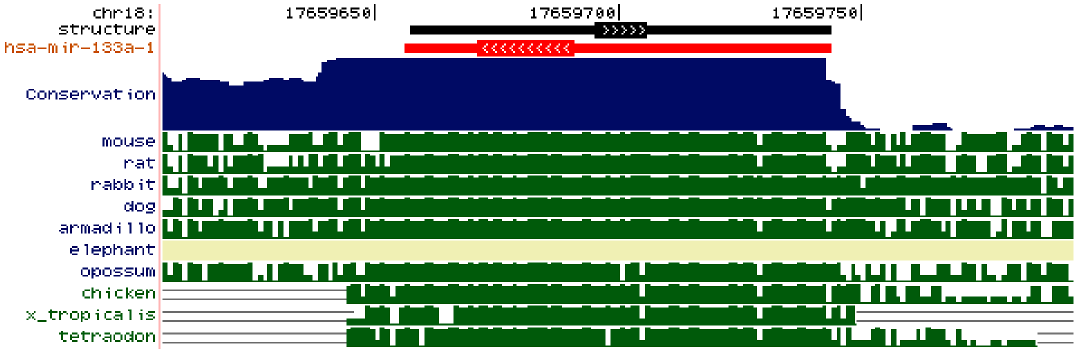

hsa-mir-181a-2   chr9:126494542-126494651 (+)

```
agaagggcuaucaggccagccuuca             A   U      CU       A    ggga
                         gaggacuccaagg ACA UCAACG  GUCGGUG GUuu    u
                         ||||||||||||| ||| ||||||  ||||||| ||||    u
                         uuccugggguuCC UGU AGUUGC  CAGUCAC CAaa    u
------------------------a             A   C      --       -    aaag
```

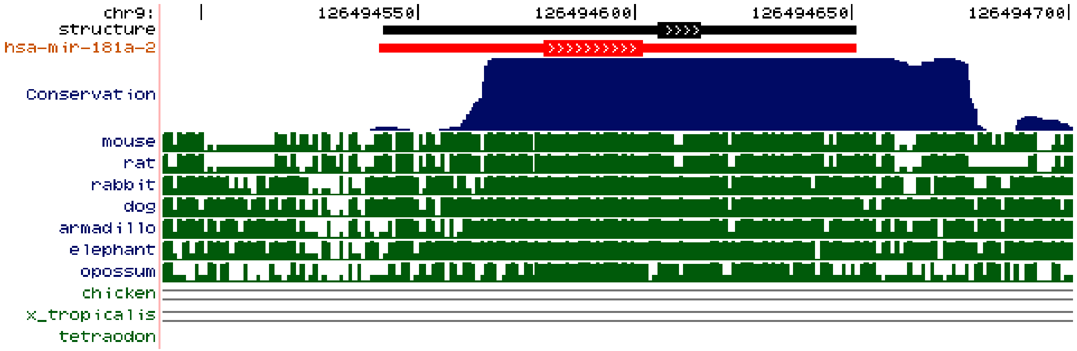

hsa-mir-125b-1   chr11:121475675-121475762 (-)

```
u    uc   u   UC  UG    C           Au     c
 gcgc  cuc cag  CC  AGA CCUAACUUGUG  guuua c
 ||||  ||| |||  ||  ||| |||||||||||  ||||| g
 cgug  gag gUC  GG  UCU GGAUUGGGCAc  uaaau u
u    cu   c   GA  GU    C           -c     u
```

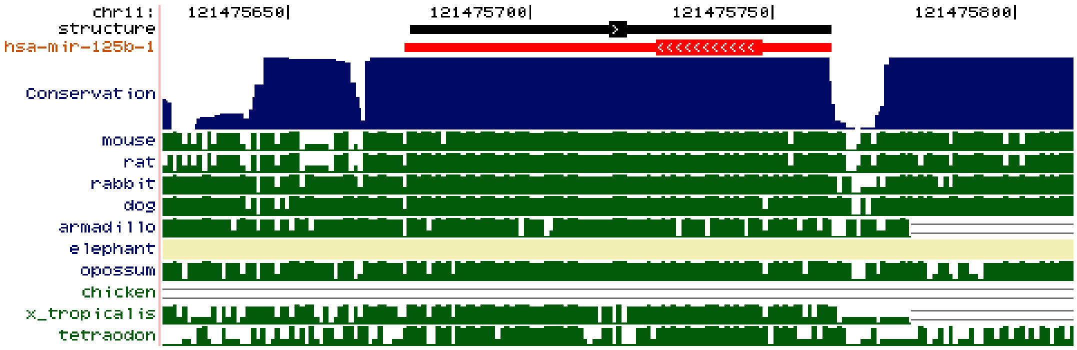

hsa-mir-582 chr5:59035189-59035286 (-)

```
----------auc  ug       aUUA                   Uaaucu
             ug  cucuuug    CAGUUGUUCAACCAGUUAC      a
             ||  |||||||    |||||||||||||||||||
             ac  gggaaac    GUCAACAAGUUGGUCAAUg      a
uuacaaagaugaa  gu       CCAA                   uuaauc
```

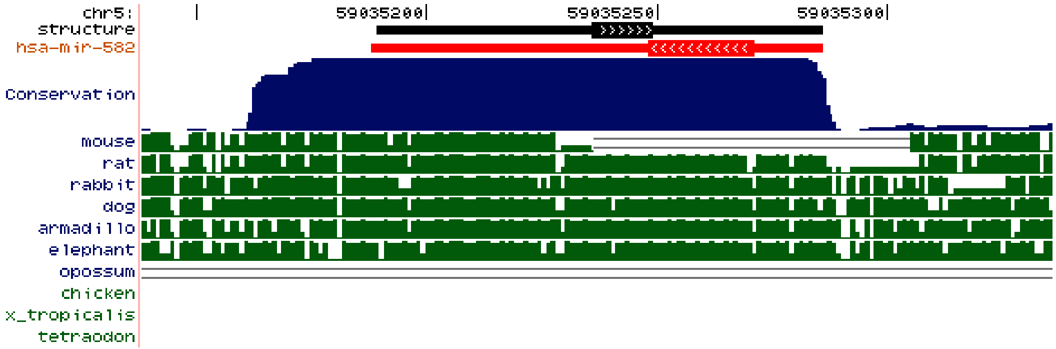

hsa-mir-18a chr13:90801006-90801076 (+)

```
u   cU    U   UC   U    A   -  aa u
 guu  AAGG GCA  UAG GCAG UAG ug  g a
 |||  |||| |||  ||| |||| ||| ||  | g
 cGG  UUCC CGU  AUC CGUC Auc ac  u a
```

```
a   UC    U   GA   C    -   u  ga u
```

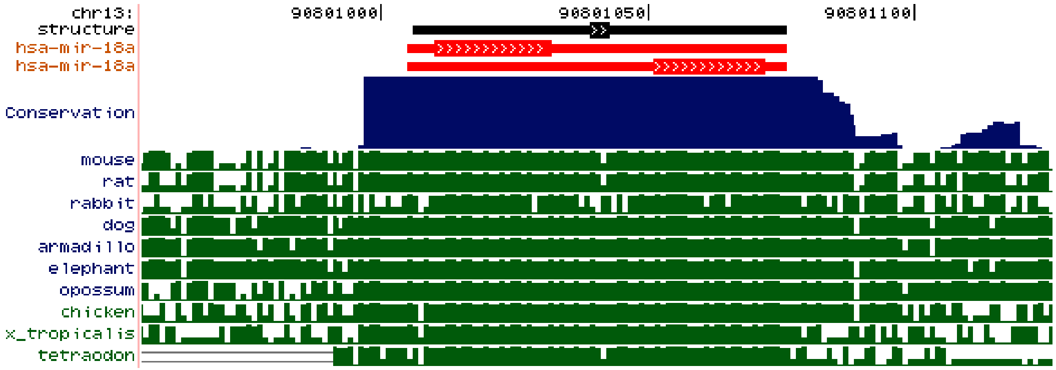

hsa-mir-103-1   chr5:167920479-167920556 (-)

```
uac    c  --    u  u           c   u g a
   ugcc uc  ggcu cu uacagugcugc uug u c u
   |||| ||  |||| || ||||||||||| ||| | |
   acgg AG  UCGG GA AUGUUACGACG Aac a g a
guu    a  UA    -  C           -   u g u
```

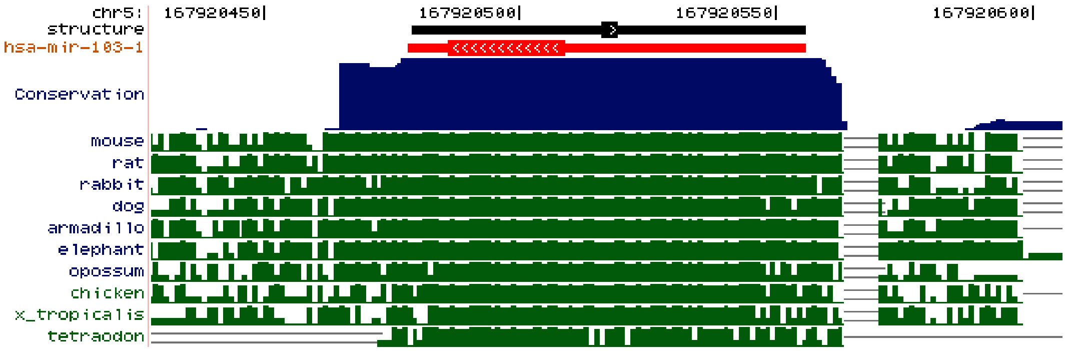

hsa-mir-148a   chr7:25956064-25956131 (-)

```
              -  -A    CC    -   ag
gaggcAAAGUUCUG AG  CACU  GACU cug  u
|||||||||||||| ||  ||||  |||| |||  a
cucUGUUUCAAGAC UC  GUGA  CUga gau  u
              A  AC    --    a   ag
```

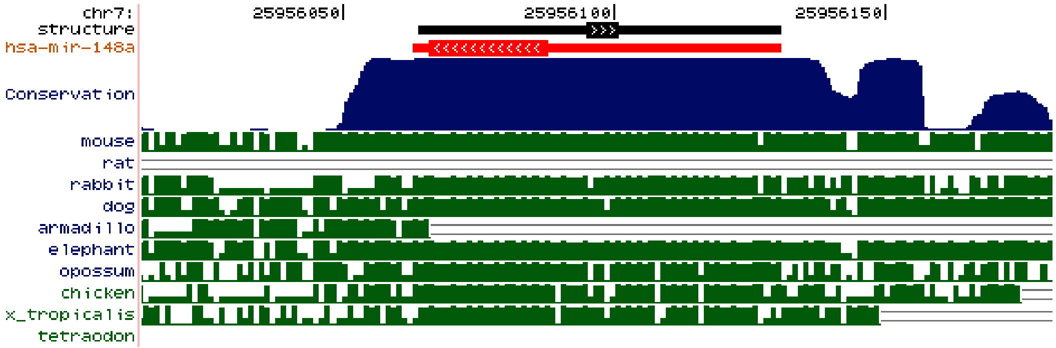

hsa-mir-19a chr13:90801146-90801227 (+)

```
    u  u                 --      ---    ag
```

```
gcag cc cuguuAGUUUUGCAUAG   UUGCAC    UACA   a
|||| || ||||||||||||||||   ||||||    ||||   a
cguc gg gguAGUCAAAACGUAUC   AACGUG    augu   g
    c  u                 UA      Uug    aa
```

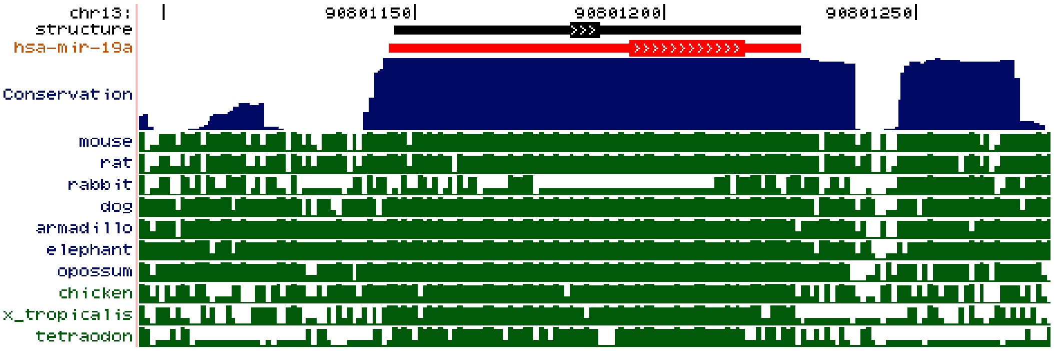

hsa-mir-1-2 chr18:17662963-17663047 (-)

```
a      c                      ac     ugaaca
 ccuacu agaguacauacuucuuuaugu  ccaua      u
 |||||| ||||||||||||||||||||  |||||
 ggaugg uuuUAUGUAUGAAGAAAUGUA  GGUau      a
c      u                      -A     cguaac
```

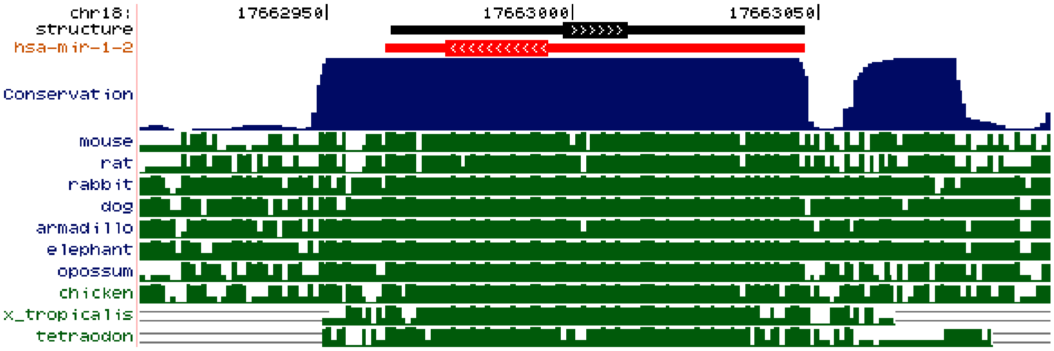

hsa-mir-30c-1   chr1:40995543-40995631 (+)

```
a     cu    ugu U       U   ACA         ---g  a
 ccaug  guag   g GUAAACA CCU   CUCUCAGCu    ug g
 |||||  ||||   | ||||||| |||   |||||||||    ||
 gguac  cguc   C CAUUUGU GGG   GAGGGUCgg    ac c
a     --    uuC U       U   --A         ugga  u
```

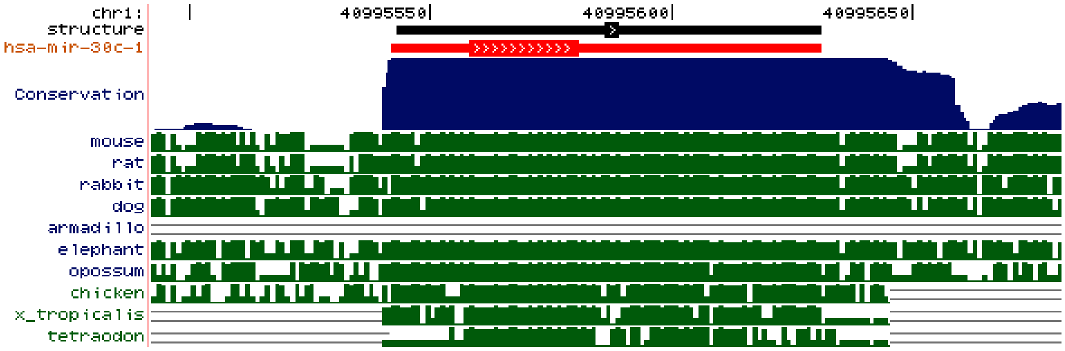

hsa-mir-140 chr16:68524485-68524584 (+)

```
u       ucucu        -  A             A       uu   uc
 gugucuc     guguccug cC GUGGUUUUACCCU UGGUAGg  acg  a
 |||||||     |||||||| || ||||||||||||| |||||||  |||
 cacgggg     cauaggac GG CACCAAGAUGGGA ACCAUcu  ugu  u
c       ----c        a  -             C       --   cg
```

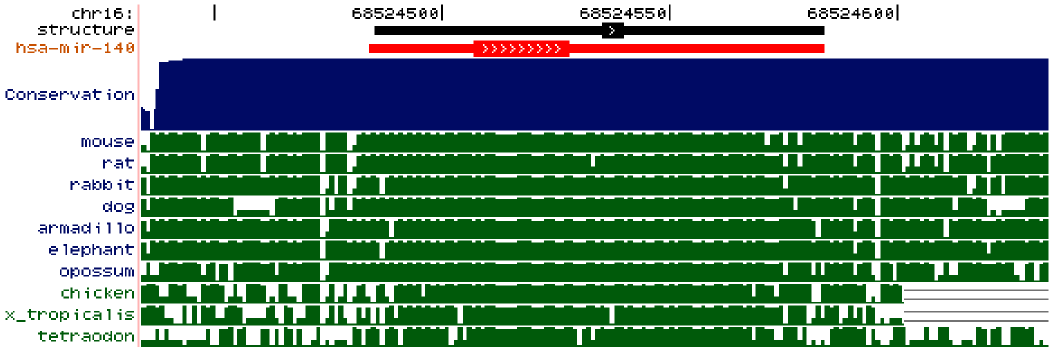

hsa-mir-382 chr14:100590396-100590471 (+)

```
    u       -A           UG      - uuu
uacu gaagaGA  GUUGUUCGUGG  GAUUCG c   a
|||| |||||||  |||||||||||  |||||| |   c
auga cuuuuuu  caacaggcacu  cuaagc g   u
    -       ca           ua      a uau
```

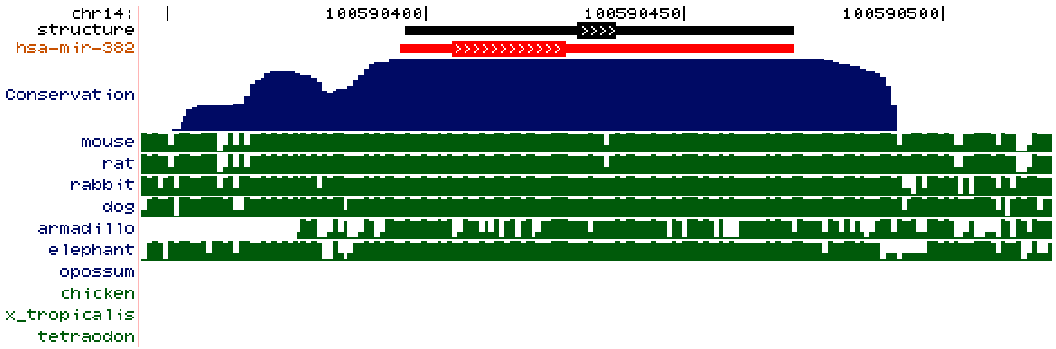

hsa-mir-323 chr14:100561822-100561907 (+)

```
---uu      u g        G U     GCGC  U    uua
     gguacu g agagAGGU G CCGUG    GU CGCu   u
     |||||| | |||||||| | |||||    || ||||
     cuauga c uuUCUCCA C GGCAC    CA gcgg   u
cuaau      - g        G U     AUUA  C    uau
```

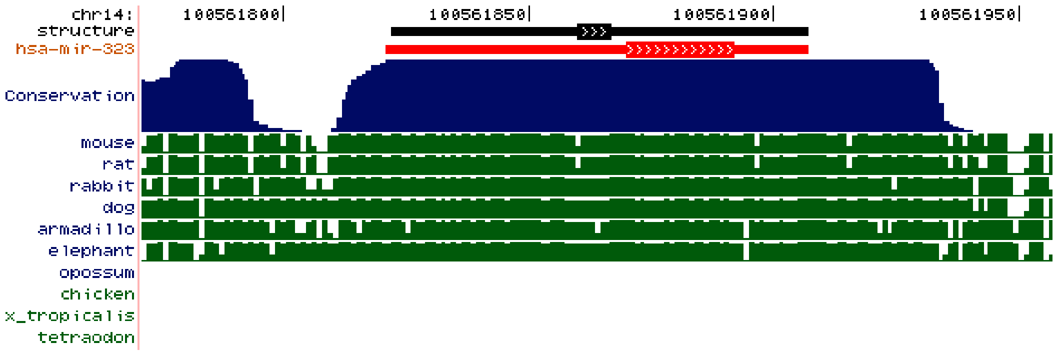

hsa-mir-34c chr11:110889374-110889450 (+)

```
     ag     A   A    A     C      C  a
agucu  uuacu GGC GUGU GUUAG UGAUUG ua u
|||||  ||||| ||| |||| ||||| |||||| ||
uuaga  aauGG CCG CACA CAAUC ACUAAc au a
     aa     A   G    C     -      c  g
```

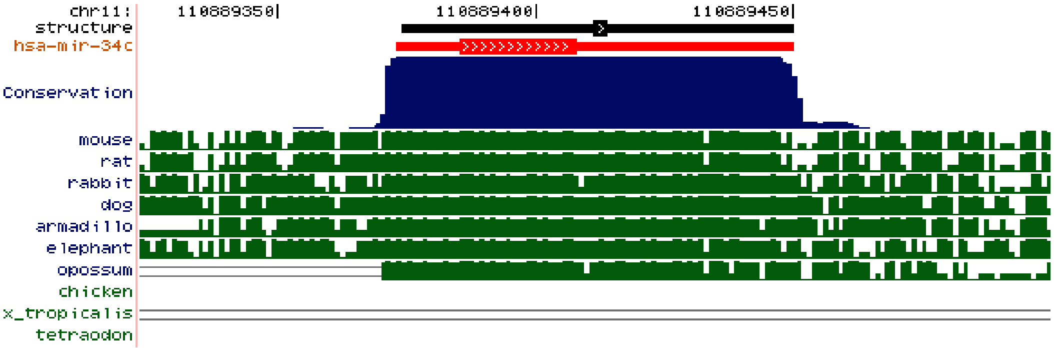

hsa-mir-96 chr7:129201768-129201845 (-)

```
ugg  g  U        A      UU       ---   uc
   cc au UUGGCACU GCACAU  UUGCUu   gug  u
   || || |||||||| ||||||  ||||||   |||
   gg UA AACCGUGA CGUGUA  AAcgag   cgc  c
aaa  G  U        -      CU       ucu   cu
```

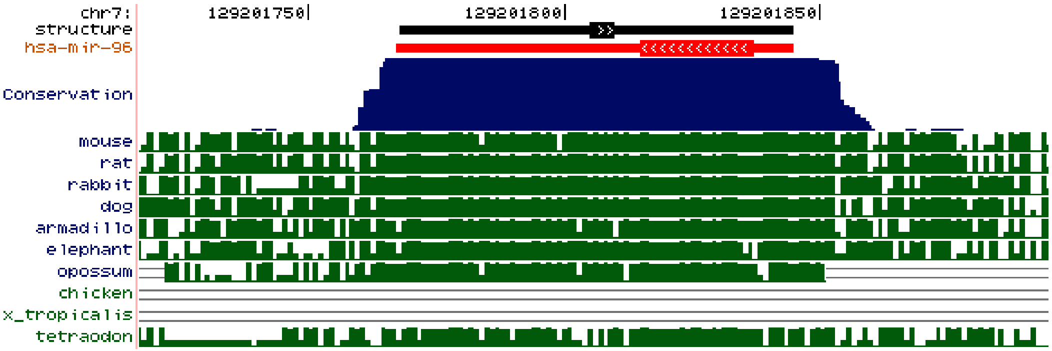

hsa-mir-9-2 chr5:87998427-87998513 (-)

```
g    c     g   UC               G      ug a
 gaag gaguu uua  UUUGGUUAUCUAGCU UAUGAg  u u
 |||| ||||| |||  ||||||||||||||| ||||||  |
 cuuc cucaa aaU  AAGCCAAUAGAUCGA AUAcuu  g u
a    -     a   GA               A      cu g
```

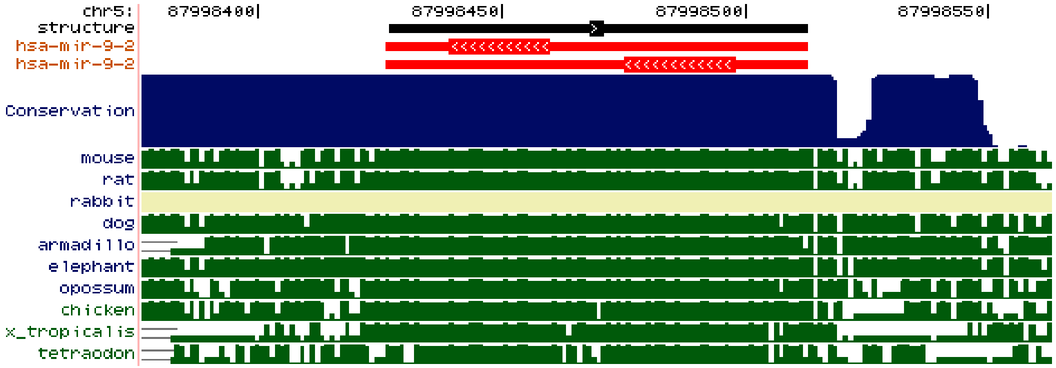

hsa-mir-9-1 chr1:154656757-154656845 (-)

```
c       guug   UC              G      u  ug
 gggguug    uua  UUUGGUUAUCUAGCU UAUGAg gg  u
 |||||||    |||  ||||||||||||||| |||||| ||  g
 ccccaau    aaU  AAGCCAAUAGAUCGA AUAcuu cu  g
a       -aaa   GA              A      -  ga
```

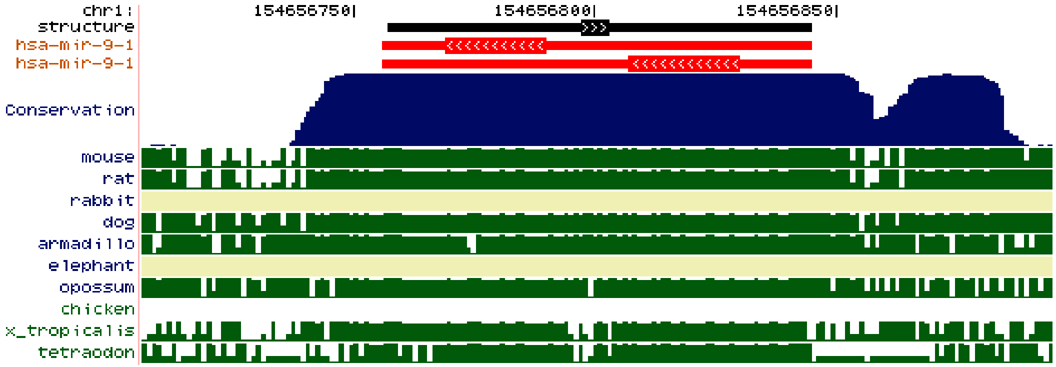

hsa-mir-876 chr9:28853624-28853704 (-)

```
u    ug         U                aucuaa  u
 gaag  cugUGGAUU CUUUGUGAAUCACCAu      gc a
 ||||  ||||||||| |||||||||||||||       ||
 cuuc  gauACUUAA GAAACAUUUGGUGGUg      ug a
a    gu         U                -----g  u
```

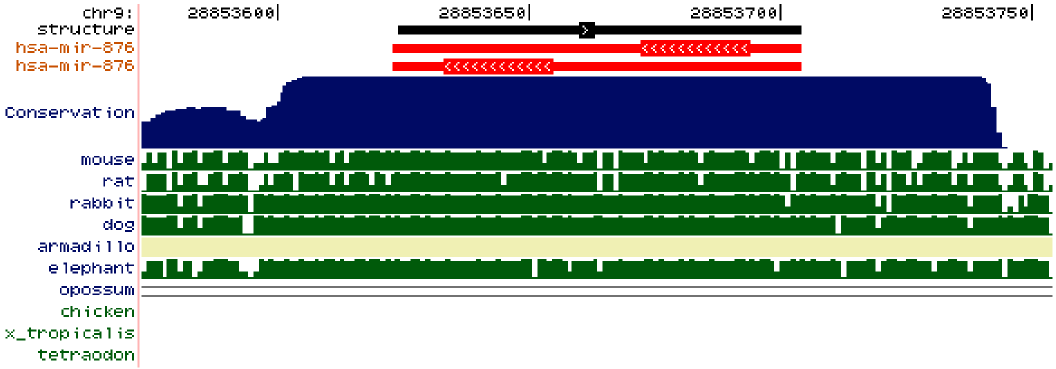

hsa-mir-873 chr9:28878877-28878953 (-)

```
--   u ca   G A     UG G        a  ga
  gug g  uuu C GGAACU  U AGUCUCCU uu  a
```

```
  ||| |  ||| | ||||||  | |||||||| ||  a
  cac c  aag g ccuuga  a ucagagga aa  a
aa   c ac    g c      gu g        c  gu
```

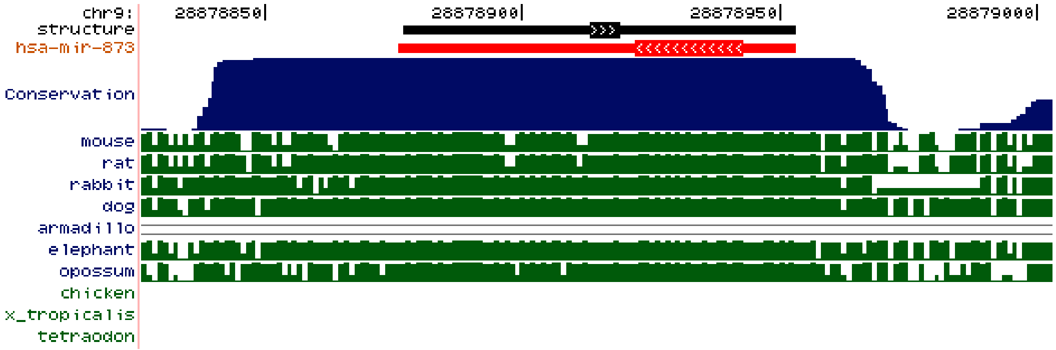

hsa-mir-7-1 chr9:85774483-85774592 (-)

```
--u    u  -      a  u        A     A       U      --     a
   ugga gu uggccu gu cugugUGG AGACU GUGAUUU GUUGUu  uuuag u
   |||| || |||||| || |||||||| ||||| ||||||| ||||||  |||||
   aucu cg accgga ca gguAUACC UCUGA CACUAAA CAACag  aaauc a
gac    c  u      -  c        G     -       -      cu     a
```

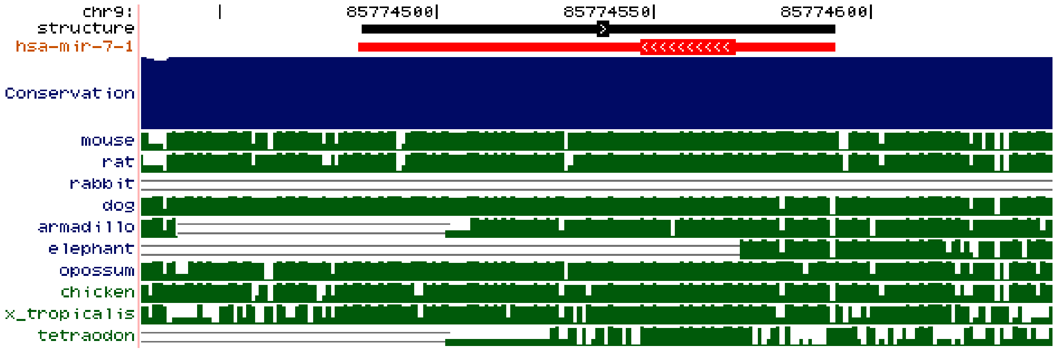

hsa-mir-592 chr7:126485378-126485474 (-)

```
---------ua     c     ca        A    C        gu    a
           uuaug cauga  UUGUGUCA UAUG GAUGAUGU  ugug u
           ||||| |||||  |||||||| |||| ||||||||  ||||
           aaugc guacu  aacgcagu gugc cuacugcg  acac g
caacacugcag     a     ac        g    a        --    g
```

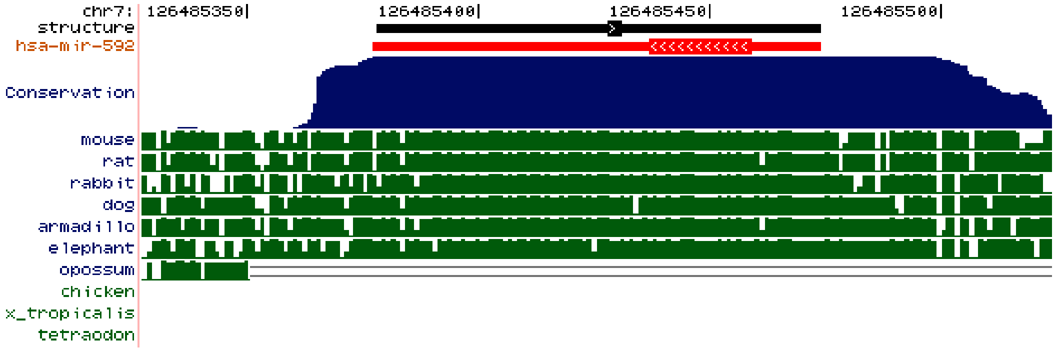

hsa-mir-423 chr17:25468223-25468316 (+)

```
-----auaa       u            -AG   G    A     cua
         aggaagu aggcUGAGGGGC   AGA CGAG CUUUu   u
         ||||||| ||||||||||||   ||| |||| |||||   u
         uccuucg ucUGACUCCCCG   UCU GCUC GAaaa   u
cgcgcccaa       u            GAG   G    -     ccu
```

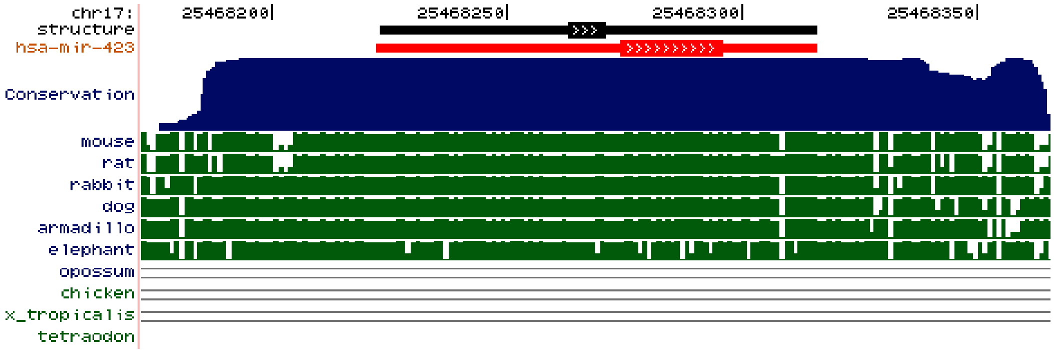

hsa-mir-411 chr14:100559415-100559510 (+)

```
--------------       ug   a a    A      A A       - uuu
              ugguacu  gag g UAGU GACCGU U GCGUACG c   a
              |||||||  ||| | |||| |||||| | ||||||| |   u
              acuauga  cuc C AUCA CUGGCA A UGUAUgc g   c
gagccccuaccuaa       --   C A    C      C A       a ugu
```

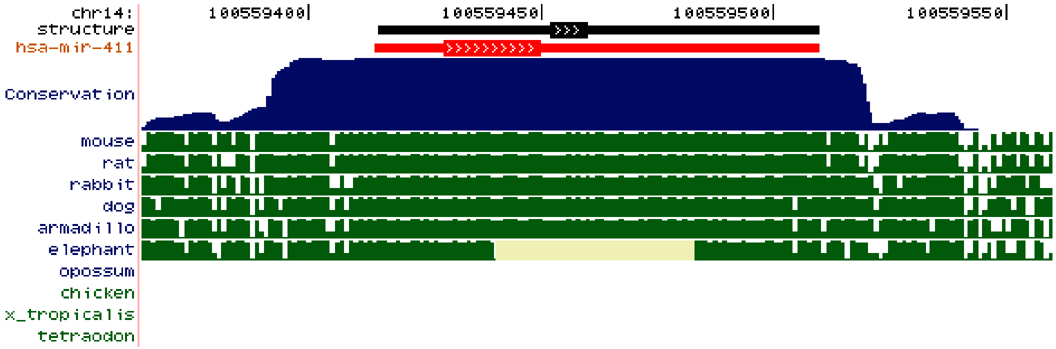

hsa-mir-383 chr8:14755318-14755390 (-)

```
   -c  A      AA    A        uug   gga
cuc  uc GAUCAG  GGUG UUGUGGCU   ggu   u
|||  || ||||||  |||| ||||||||   |||
gag  ag cugguc  ucac gacaccga   cua   a
   aa  a      cg    -        ---   auu
```

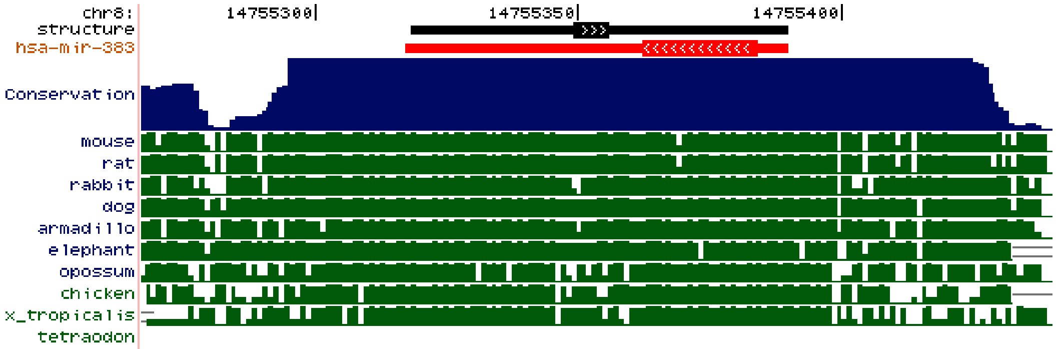

hsa-mir-379 chr14:100558156-100558222 (+)

```
    a    A       GA       -  uu u
agag UGGU GACUAUG  ACGUAGG cg  a g
|||| |||| |||||||  ||||||| ||  |
ucUC AUCA CUGGUAC  UGUAUcc gu  u a
    A    C       AA       a  cu u
```

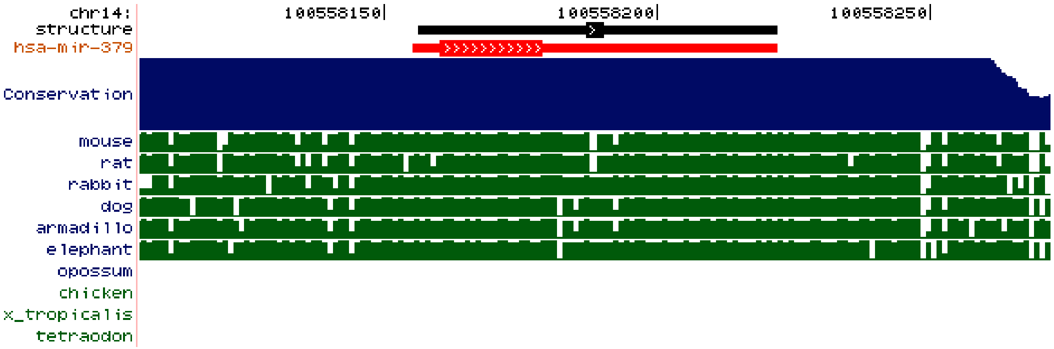

hsa-mir-378 chr5:149092581-149092646 (+)

```
   g  C                UGU      ccu
agg CU CUGACUCCAGGUCC   GUguua   a
||| || ||||||||||||||   ||||||
ucc GA GACUGAGGUUCAGG   CAcgau   g
   G  A                --U      aaa
```

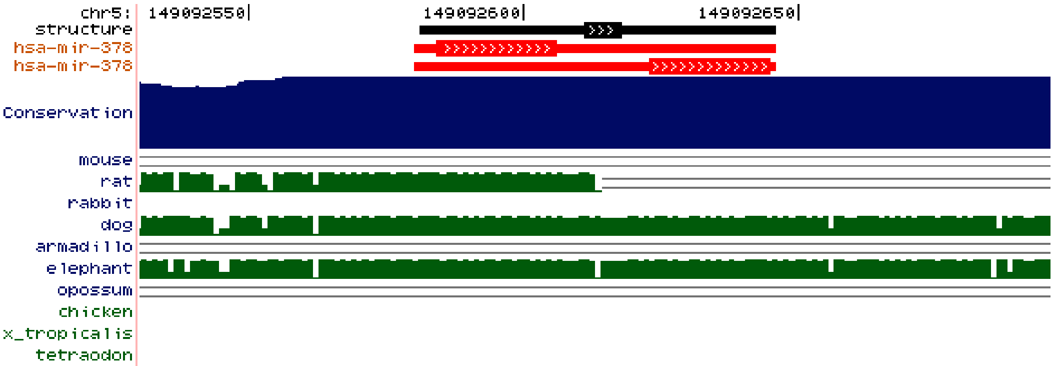

hsa-mir-377 chr14:100598140-100598208 (+)

```
uu              C   -    A    - uuu
  gagcAGAGGUUGCC UUG GUGA UUCg c   a
  |||||||||||||| ||| |||| |||| |   u
  uuUGUUUUCAACGG AAC CACU Aagu g   u
-g              A   A    -    u uau
```

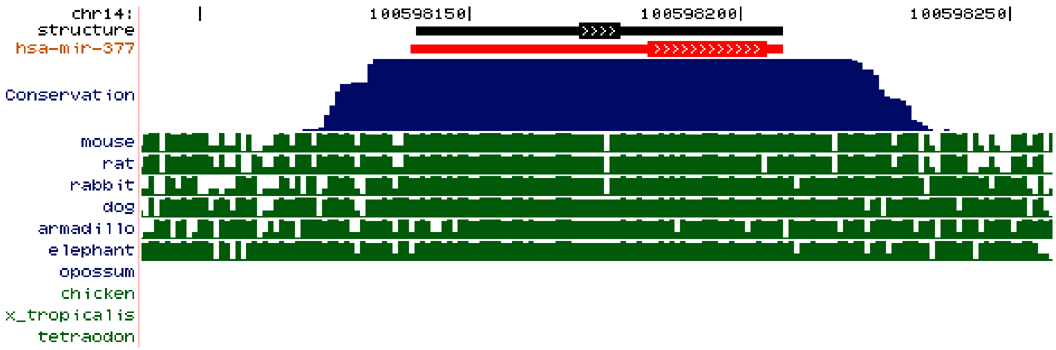

hsa-mir-376a-1   chr14:100576872-100576939 (+)

```
u    g  A    C    U      --GUA   u
 aaaa GU GAUU UCCU CUAUGA     cau a
 |||| || |||| |||| ||||||     ||| u
 uuuU CA CUAA AGGA GAUACU     gua u
c    G  C    A    -      Aauua   u
```

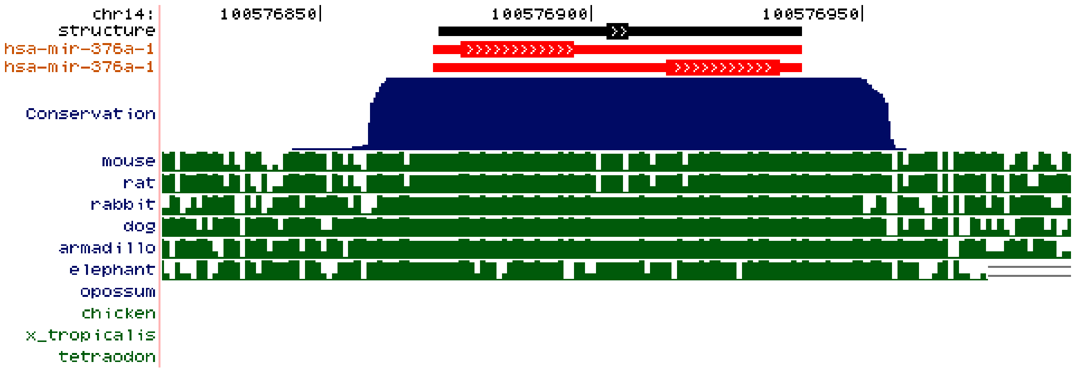

hsa-mir-375 chr2:219574611-219574674 (-)

```
c  -    a      ccu  c     c  gac
 cc cgcg cgagcc   cg acaaa cg   c
 || |||| ||||||   || ||||| ||
 gg GUGC GCUCGG   GC UGUUU gc   u
c  A    -      CUU  U     u  gag
```

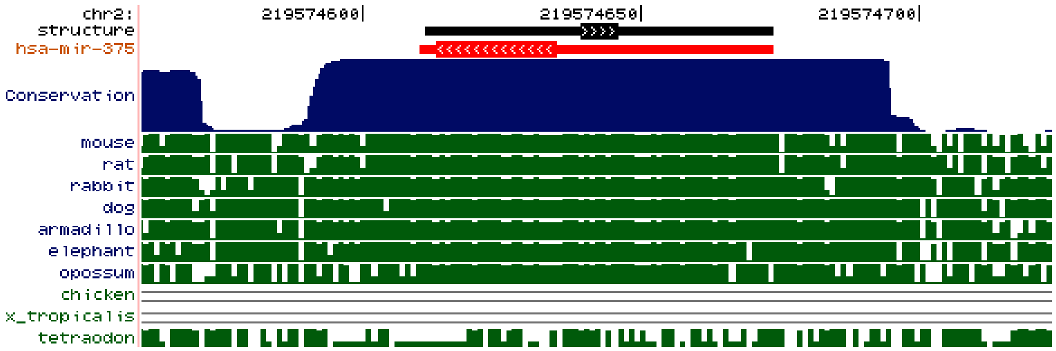

hsa-mir-365-1   chr16:14310643-14310729 (+)

```
acc      aa        ac            ----   -   uuu
   gcaggg  aaugaggg  uuuugggggca    gau gug   c
   ||||||  ||||||||  |||||||||||    ||| |||   c
```

```
   cguucu  uUAUUCCU  AAAAUCCCCGU    cua cac   a
--a      cg        -A           AAUa   u   cuu
```

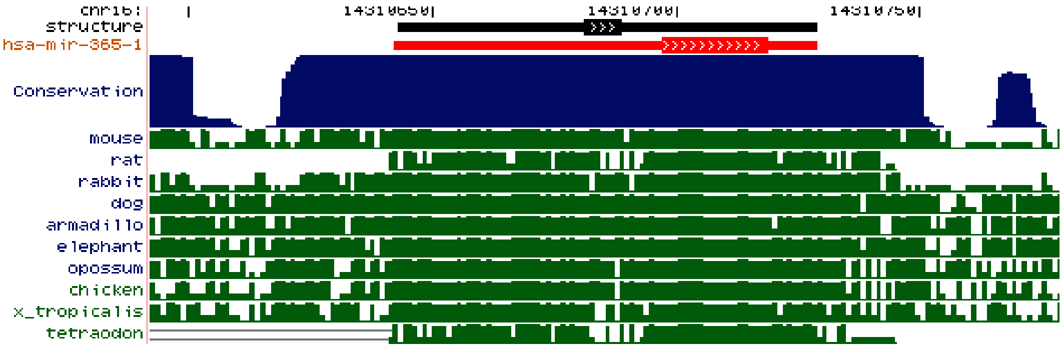

hsa-mir-331 chr12:94226327-94226420 (+)

```
gaguuugguuuu         u        U    U       AU Ccaga
            guuuggguu guuCUAGG AUGG CCCAGGG  C     u
            ||||||||| |||||||| |||| |||||||  |     c
            cgaauccaa cAAGAUCC UAUC GGGUCCC  g     a
----------cu         c        -    C       CG accaa
```

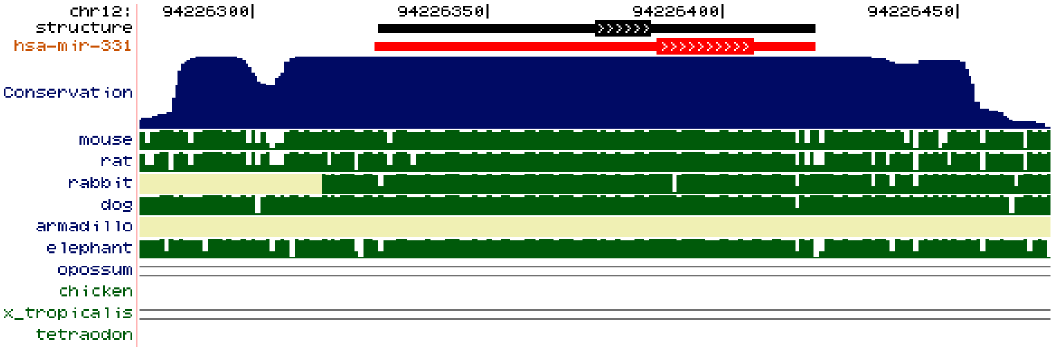

hsa-mir-32 chr9:110848330-110848399 (-)

```
        UG      U           - uu  c
ggagaUAU  CACAU ACUAAGUUGCAu g  gu a
||||||||  ||||| |||||||||||| |  ||
cuUUUAUA  GUGUG UGAUUUAACgua c  cg c
        GU      -           a uc  g
```

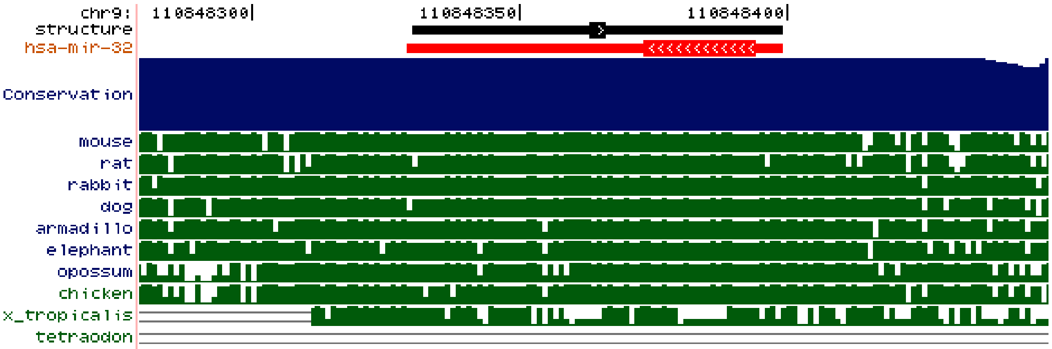

hsa-mir-31 chr9:21502114-21502184 (-)

```
      gA      G   C         -U    gaa
```

```
ggagag  GGCAA AUG UGGCAUAGC  guu   c
||||||  ||||| ||| |||||||||  |||
ccuuuc  CCGUU UAC ACCGUAUCG  caa   u
      UA     A   A         Uc   ggg
```

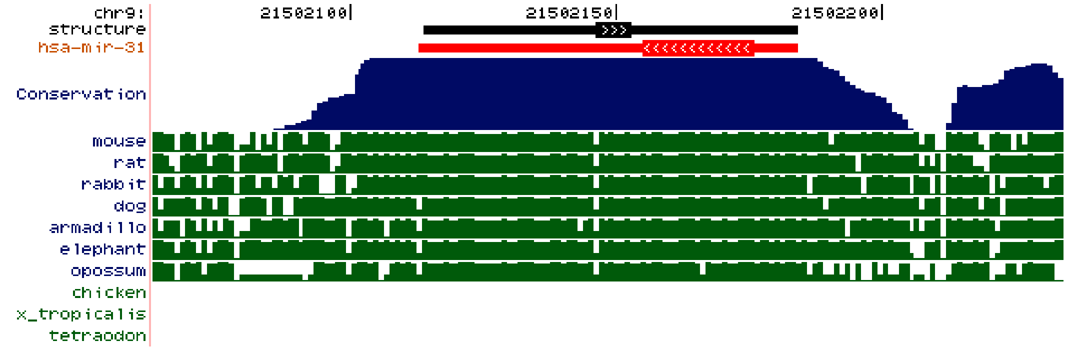

hsa-mir-29c chr1:206041820-206041907 (-)

```
a         -  ggc          UCC       ---     u
 ucucuuaca ca   UGACCGAUUUC   UGGUGUU   Cagag c
 ||||||||| ||   |||||||||||   |||||||   ||||| u
 gggggaugu gu   AUUGGCUAAAG   ACCACGA   guuuu g
a         a  ---          UUU       Ucu     u
```

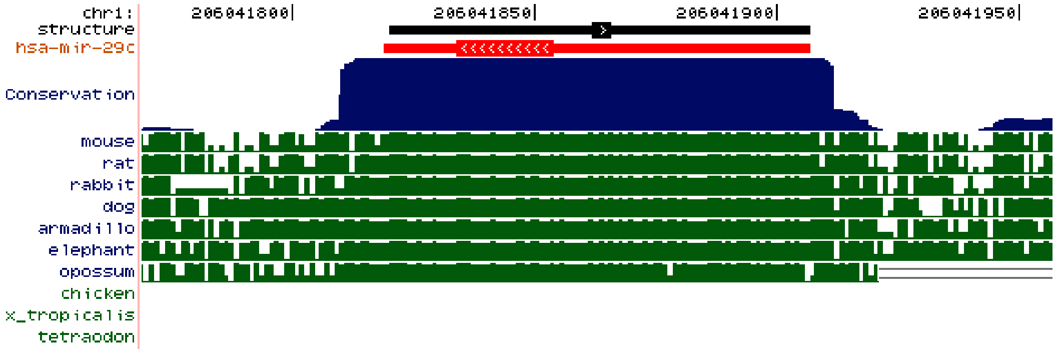

hsa-mir-29b-2   chr1:206042411-206042491 (-)

```
         -          C      G  U    uuuucc
cuucuggaa gCUGGUUUCA AUGGUG CU AGau      a
||||||||| |||||||||| |||||| || ||||
gaggauuUU UGACUAAAGU UACCAC GA Ucua      u
         G          U      -  -    uguuuc
```

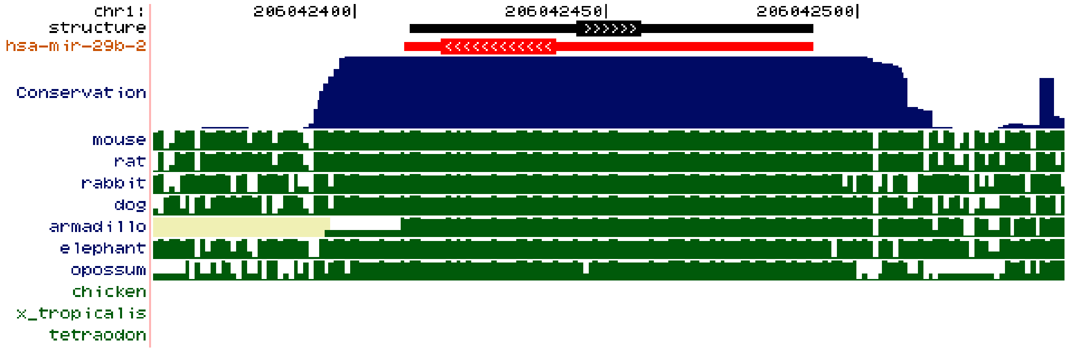

hsa-mir-29b-1   chr7:130212758-130212838 (-)

```
-         -          U      GU      uuaaa
 cuucaggaa GCUGGUUUCA AUGGUG  UUAGAu     u
 ||||||||| |||||||||| ||||||  ||||||     a
 gggguucUU UGACUAAAGU UACCAC  GAUcug     g
g         G          U      --      uuagu
```

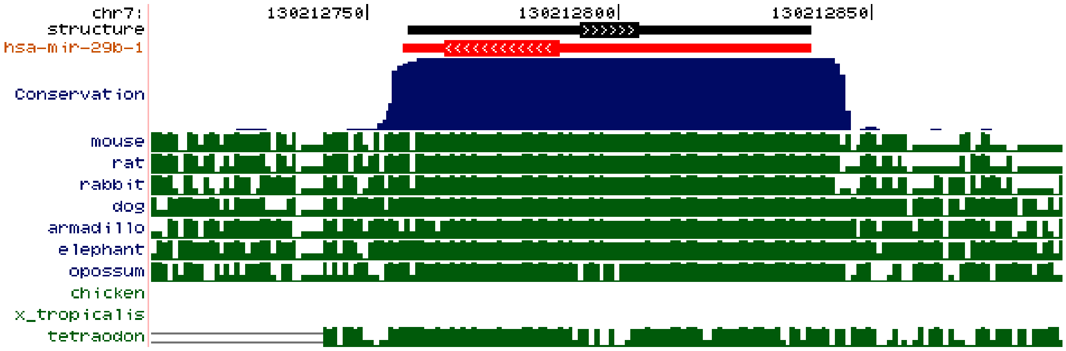

hsa-mir-29a chr7:130212046-130212109 (-)

```
            UUU       C    ucaa
augACUGAUUUC   UGGUGUU AGag    u
||||||||||||   ||||||| ||||    a
uAUUGGCUAAAG   ACCACGA Ucuu    u
            UCU       -    uuaa
```

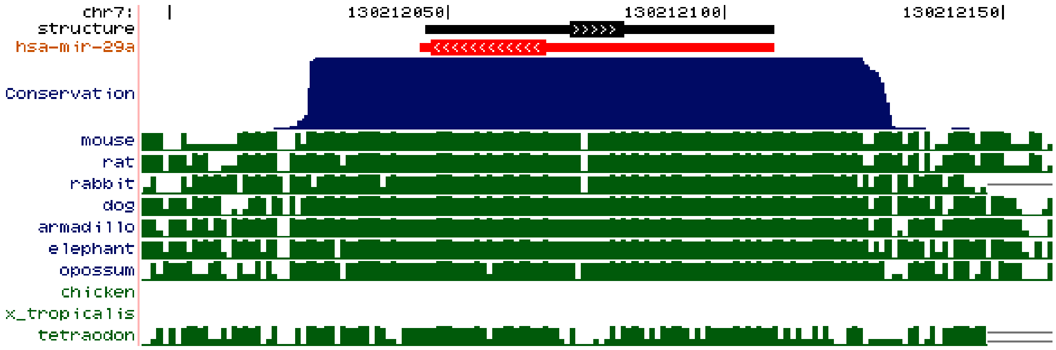

hsa-mir-25 chr7:99529119-99529202 (-)

```
    a ug   ag    G     UU G     U   --  ac
ggcc g  uug  AGGC GAGAC  G GCAAU Gcu  gg  g
|||| |  |||  |||| |||||  | ||||| |||  ||  c
ccgg c  gac  UCUG CUCUG  C CGUUA Cgg  cc  u
    c gu   AG    G     UU A     -   gu  cg
```

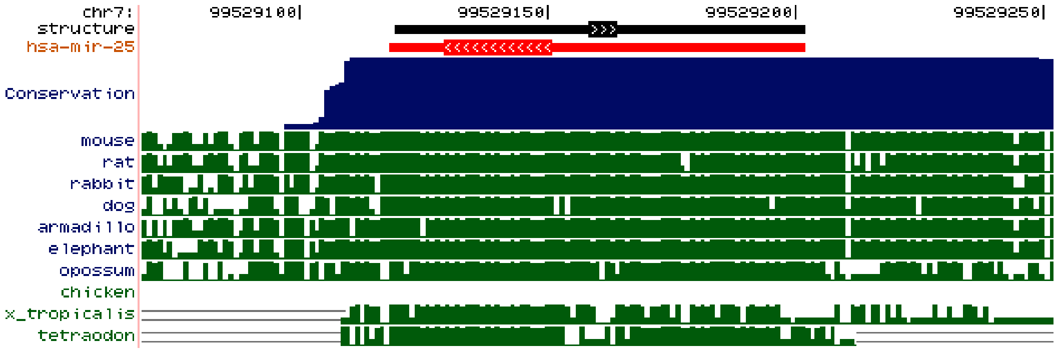

hsa-mir-22 chr17:1563947-1564031 (-)

```
   u   cc               -     A      u  ccu
ggc gag  gcaguAGUUCUUCAG UGGCA GCUUUA gu   g
||| |||  ||||||||||||||| ||||| |||||| ||   a
ccg cuc  cguUGUCAAGAAGUU ACCGU CGAAau cg   c
   u   -c               G     -      -  acc
```

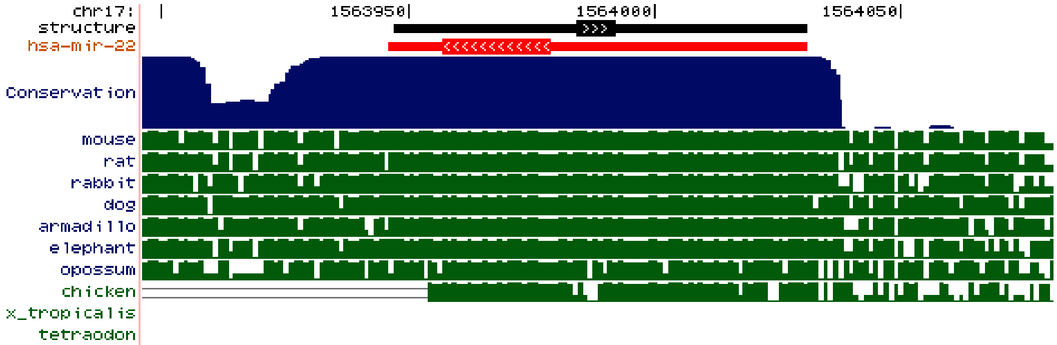

hsa-mir-21 chr17:55273409-55273480 (+)

```
u     gU        A     A     A    u a
 gucgg  AGCUUAUC GACUG UGUUG cugu g a
 |||||  |||||||| ||||| ||||| |||| | u
 caguc  UCGGGUAG CUGAC ACAAC ggua c c
a     UG        -     C     -    - u
```

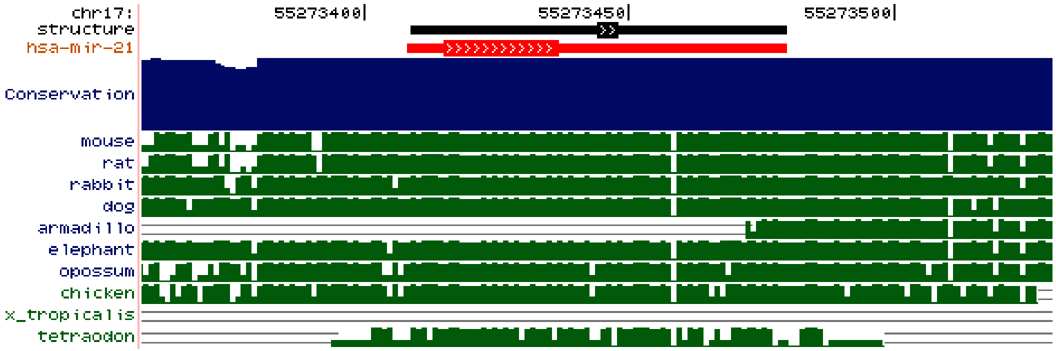

hsa-mir-204 chr9:72614711-72614820 (-)

```
ggcuacagucuuucu   -  -    ucg     U          A     U    gagaau
               uca ug ugac   uggac UCCCUUUGUC UCCUA GCCU      a
               ||| || ||||   ||||| |||||||||| ||||| ||||      u
               ggu ac acug   acuug agggaaacgg agggu cgga      a
--------------c   c  u    uua     c          a     -    ggaagu
```

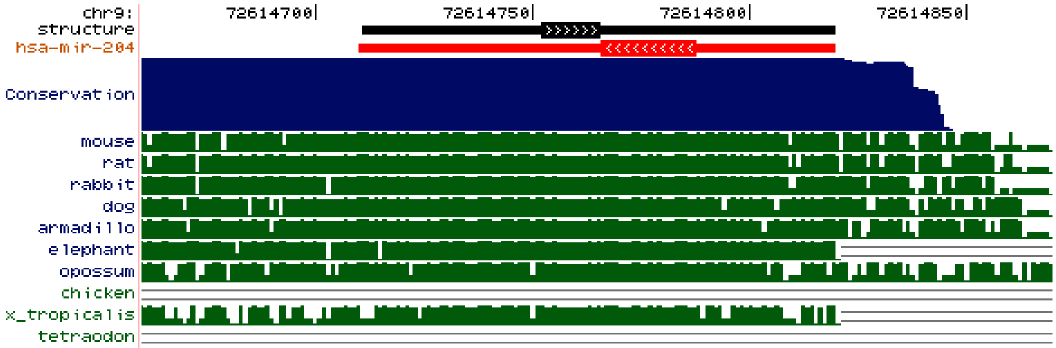

hsa-mir-196b   chr7:27175624-27175707 (-)

```
--     uc      uU       U  C            ucca
  acugg  ggugau  AGGUAGU UC UGUUGUUGGGa    c
  |||||  ||||||  ||||||| || |||||||||||
  ugacu  ucauua  uccguca ag acgacagcucu    c
gu     --      cu       c  c            cuuu
```

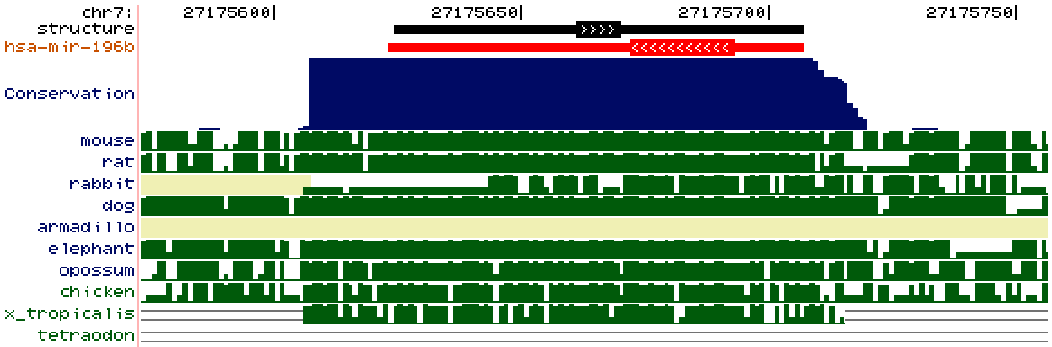

hsa-mir-193b   chr16:14305325-14305407 (+)

```
   gu       u     GU         G  A  Ag uua
gug  cucagaa CGGG   UUUGAGGGC AG UG  u   u
|||  ||||||| ||||   ||||||||| || ||  |   g
uac  ggguuuU GCCC   AAACUCCCG UC Ac  a   u
   ug       C     UG         G  A  cu uuu
```

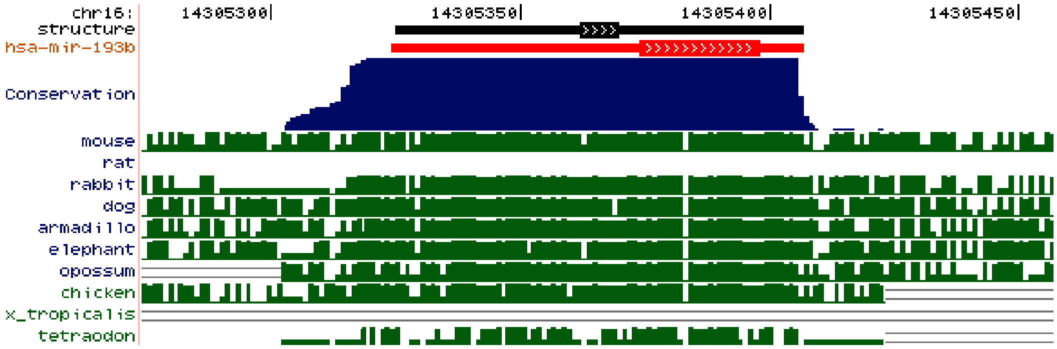

hsa-mir-190 chr15:60903209-60903293 (+)

```
u     c u    - UG               UA      uuau
 gcagg c cugu g  AUAUGUUUGAUAUAU  GGUug    u
 ||||| | |||| |  |||||||||||||||  |||||
 cguuc g gaca c  uauacaaacuauaua  ucaac    u
```

```
c     u u    u cu              --      cuaa
```

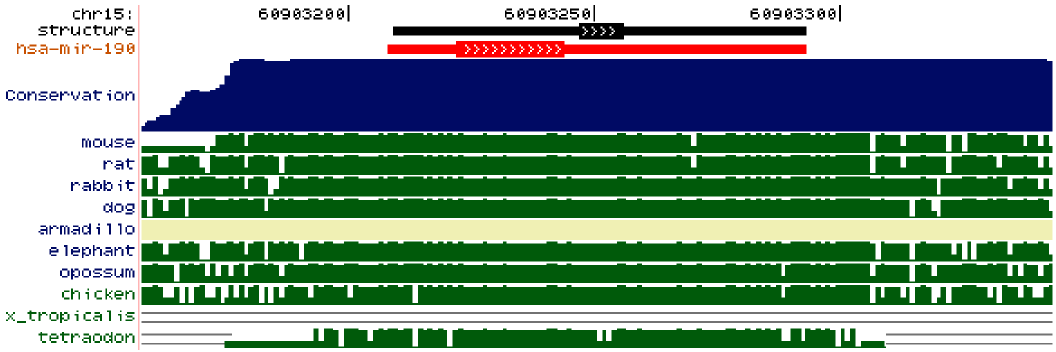

hsa-mir-181b-1   chr1:197094625-197094734 (-)

```
ccugugcagagauuauuuuuuaaaa       aucAA       CUG          gaa  g
                         ggucaca     CAUUCAUUG   UCGGUGGGUu   cu u
                         |||||||     |||||||||   ||||||||||   ||
                         ccggugu     guaaguaac   agucacucga   gg g
-------------------uucgcc       -caac       --a          aca  u
```

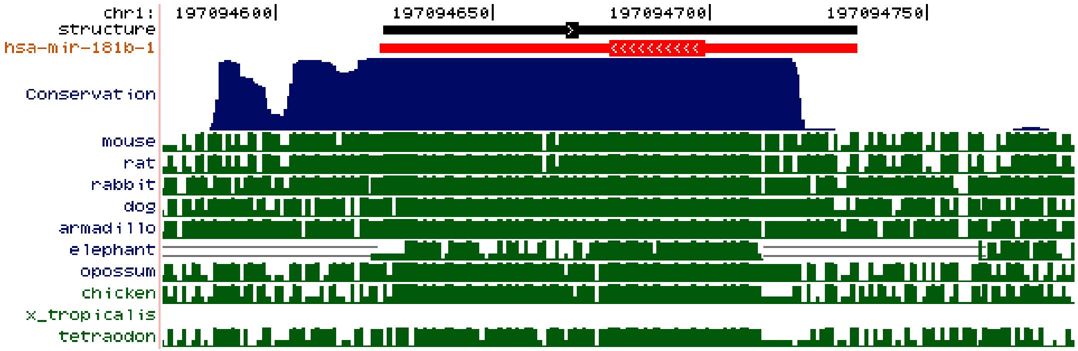

hsa-mir-153-2   chr7:157059789-157059875 (-)

```
a     gg      -             gu       -a  aa
 gcggu  ccagug ucauuuuugugau  ugcagcu  gu  u
 |||||  |||||| |||||||||||||  |||||||  ||  a
 uguca  gguuaC AGUGAAAACACUG  ACGUUga  cg  u
g     aa      U             AU       cc  ag
```

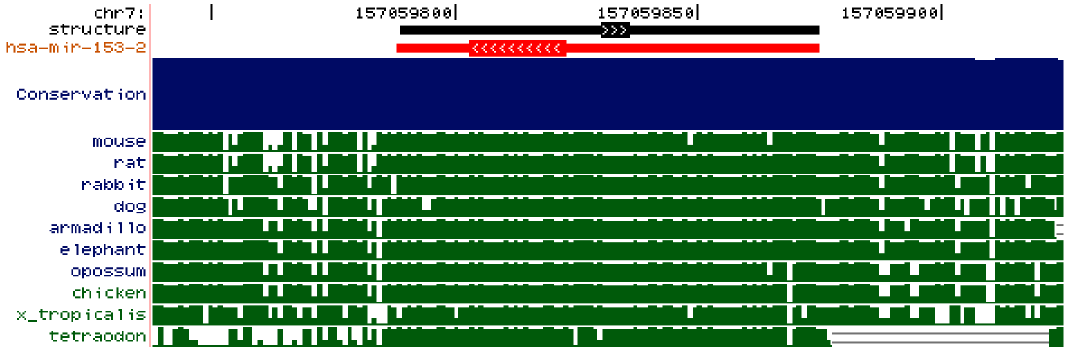

hsa-mir-142 chr17:53763592-53763678 (-)

```
g        g   c           A         Uaa ag a
 acagugca uca cCAUAAAGUAG AAGCACUAC   c  c c
```

```
 |||||||| ||| ||||||||||| |||||||||   |  |
 ugucaugu agu GGUAUUUCAUC UUUGUGAUG   g  g u
g        g   A           C         -Ug ga g
```

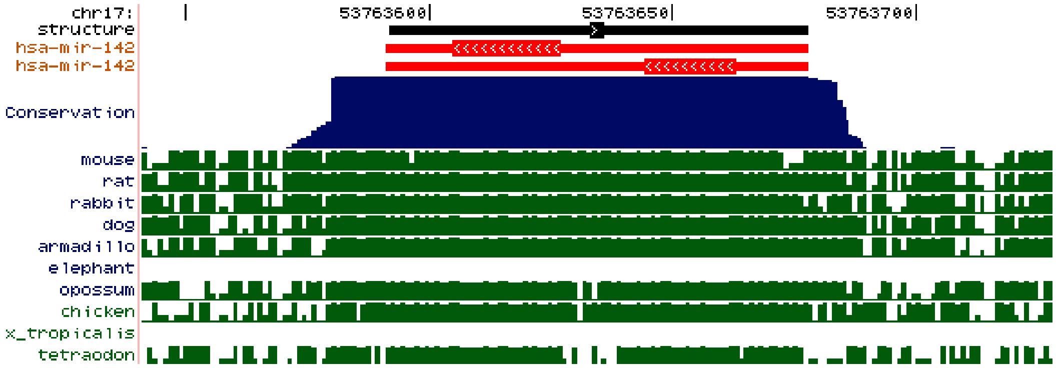


```
hsa-mir-138-2     chr16:55449931-55450014 (+)

c u   u  AG              UCA     -ac   c  cg
 g ugc gc  CUGGUGUUGUGAA   GGCCG   gag ag  c
 | ||| ||  |||||||||||||   |||||   ||| ||
 c acg UG  GACCACAGCACUU   UCGgc   uuc uc  a
a u   U -G              -UA     cca   -  cu
```

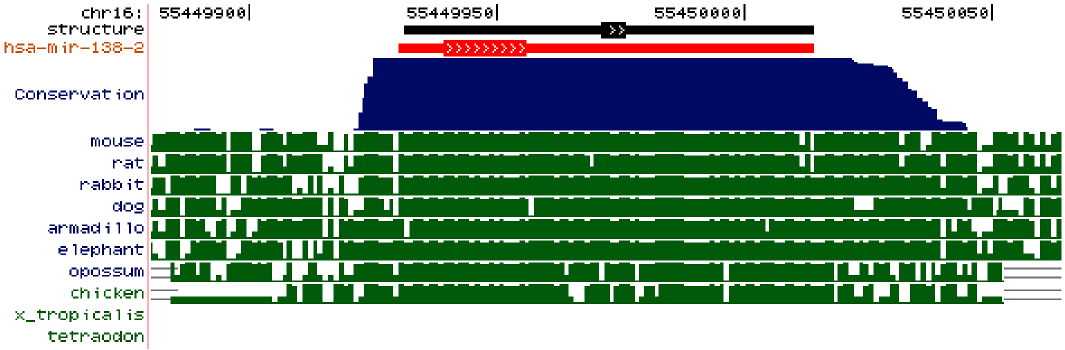


```
hsa-mir-135a-2    chr12:96481721-96481820 (+)

a   aaa     ucua   c            U            u g a
 gau   uucac    gug uuUAUGGCUUUU AUUCCUAUGUGA a u a
 |||   |||||    ||| |||||||||||| |||||||||||| | | u
 cua   aagug    cau aaguaccgaagg uagggauguacu u a a
a   -aa     -uua   a            -            c g a
```

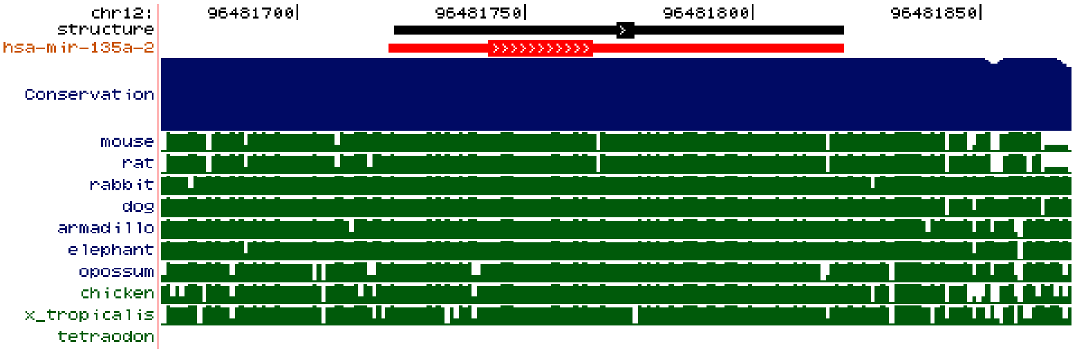


```
hsa-mir-128a      chr2:136139437-136139518 (+)
```

```
u    u      uuc       uag      cu       u
 gagc guugga   ggggccg   cacugu  gagaggu u
 |||| ||||||   |||||||   ||||||  |||||||
 uucg cgacuu   CUCUGGC   GUGACA  CUcuuua a
c    u      UUU       CAA      --       c
```

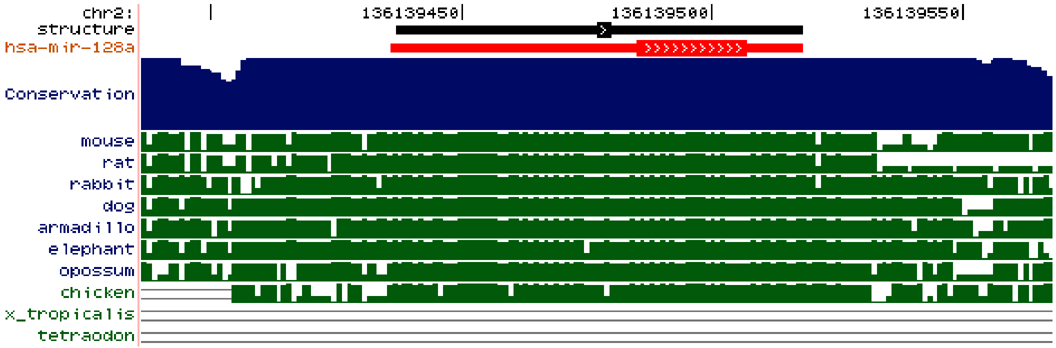

hsa-mir-107 chr10:91342484-91342564 (-)

```
c   c      --c   u  u           c   u   a
 ucu ugcuuu   agcu cu uacaguguugc uug ggc u
 ||| ||||||   |||| || ||||||||||| ||| ||| g
 aga acgaaA   UCGG GA AUGUUACGACG Aac uug g
-   c      CUA   -  C           -   -   a
```

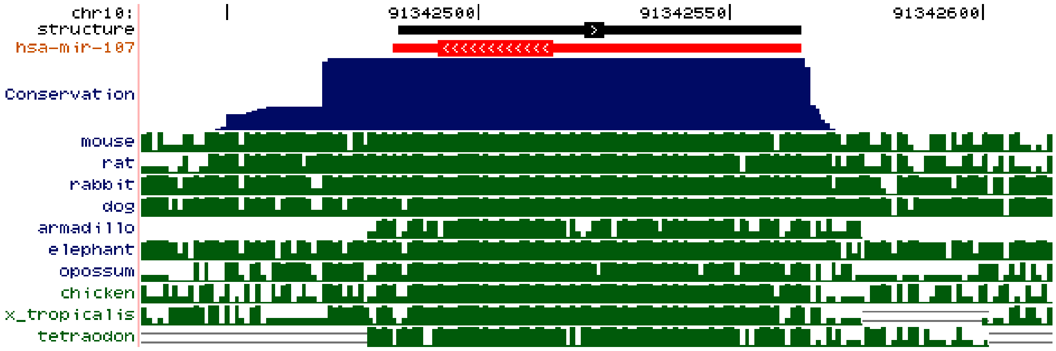

hsa-mir-101-1   chr1:65296705-65296779 (-)

```
u   cuggc                  A    gucua
 gcc     uCAGUUAUCACAGUGCUG UGCU     u
 |||     |||||||||||||||||| ||||
 cgg     AGUCAAUAGUGUCAUGAC AUgg     u
a   uaggA                  -    aaauc
```

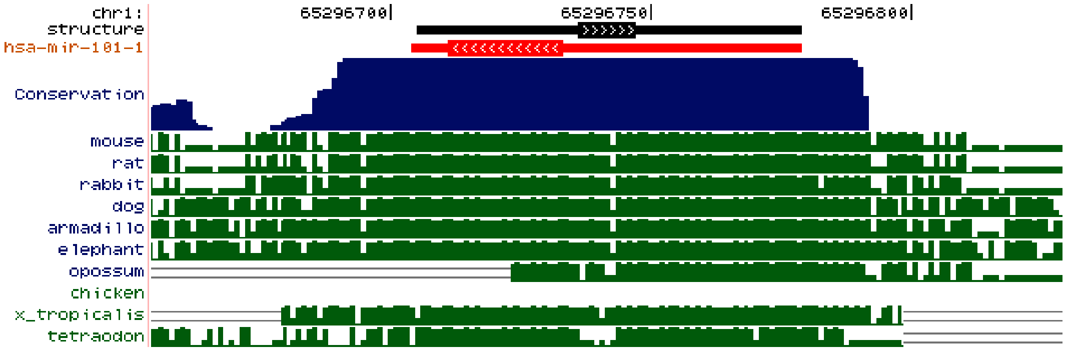

hsa-let-7i chr12:61283733-61283816 (+)

```
c    U                 U   --------  u     ugu
 uggc GAGGUAGUAGUUUGUGC GUU        gg cgggu   g
 |||| ||||||||||||||||| |||        || |||||   a
 aUCG UUCCGUCAUCGAACGCG Caa        uc gcccg   c
-    -                 U   uagaggug  -     uua
```

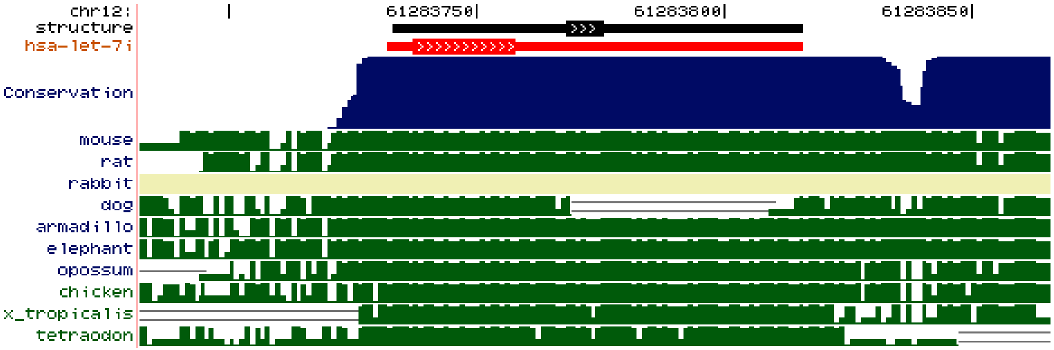

hsa-let-7g chr3:52277334-52277417 (-)

```
a   U        A            ugagg    -a  a    a
 ggc GAGGUAGU GUUUGUACAGUU     gucu  ug uacc c
 ||| |||||||| ||||||||||||     ||||  || ||||
 cCG UUCCGUCA CGGACAUGUCaa     uaga  ac augg c
a   -        C            -----    gg  -    c
```

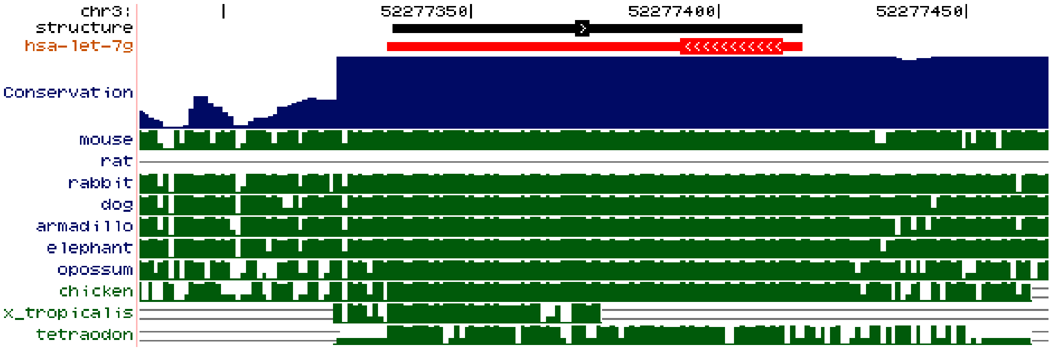

hsa-let-7f-2   chrX:53600878-53600960 (-)

```
u      U   GU                uuagggucauac
 guggga GAG  AGUAGAUUGUAUAGUU            c
 |||||| |||  ||||||||||||||||
 cacCCU UUC  UCAUCUGACAUAUCaa            c
g      -   UG                uagagguucuac
```

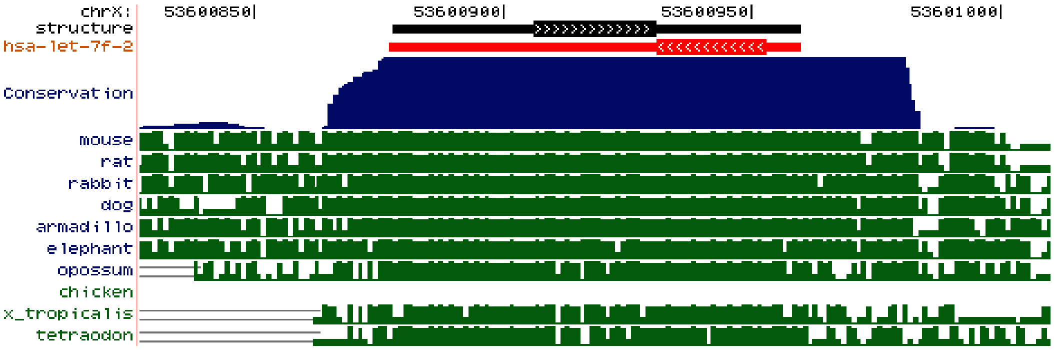

hsa-let-7f-1   chr9:95978450-95978536 (+)

```
    a UG                      ---------       u
ucag g  AGGUAGUAGAUUGUAUAGUUgu         gggguag g
|||| |  ||||||||||||||||||||||         ||||||| a
aguC C  UCCGUUAUCUAACAUAUCaaua         ucccauu u
    - CU                      gaggacuug       u
```

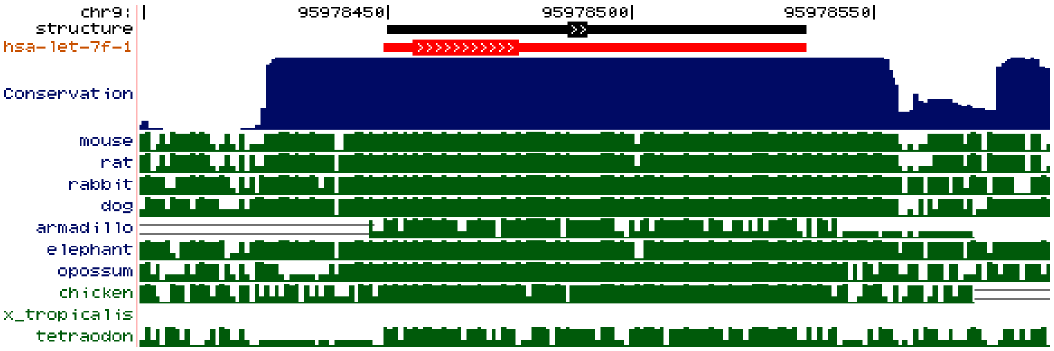

hsa-let-7d chr9:95980937-95981023 (+)

```
       A               C      uuagggcagggauu
ccuagga GAGGUAGUAGGUUG AUAGUU              u
||||||| |||||||||||||| ||||||
ggauUCU UUCCGUCGUCCAGC UAUCaa              u
       -               A      uggaggaacacccg
```

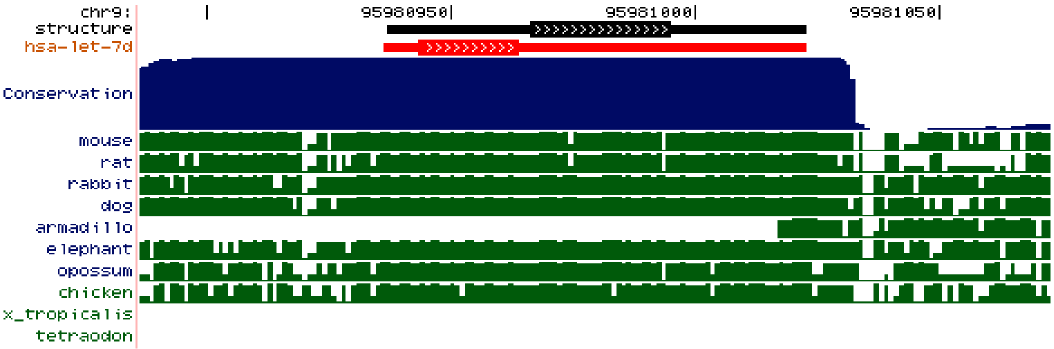

hsa-let-7a-2   chr11:121522440-121522511 (-)

```
   uU   G   U             uagaa ua a
agg  GAG UAG AGGUUGUAUAGUU     u  c u
|||  ||| ||| |||||||||||||     |  | c
ucc  uuc auc uccgacaugucaa     a  g a
```

```
   -u   g   c             --uag gg a
```

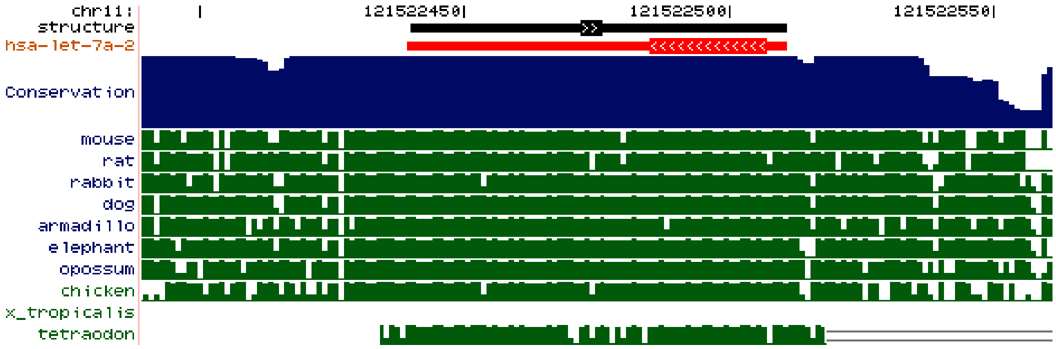

hsa-let-7a-1   chr9:95978060-95978139 (+)

```
     U   GU                 uuagggucacac
uggga GAG  AGUAGGUUGUAUAGUU            c
||||| |||  ||||||||||||||||            c
aucCU UUC  UCAUCUAACAUAUCaa            a
     -   UG                 uagagggucacc
```

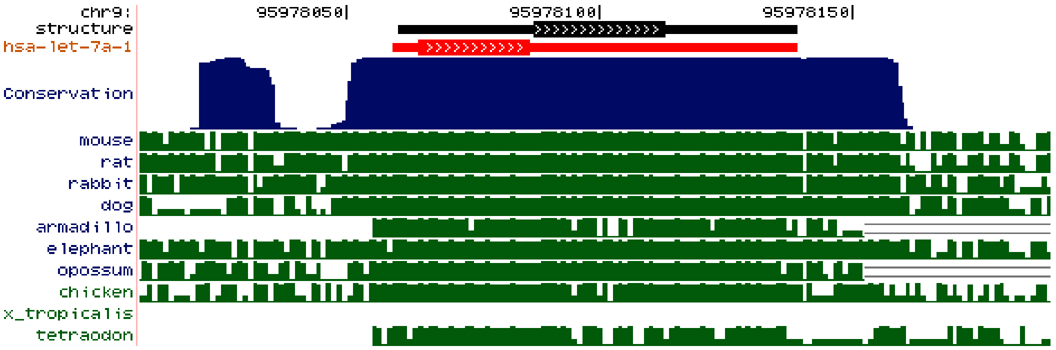

hsa-mir-30c-2   chr6:72143384-72143455 (-)

```
   uacU       U   ACA         guggaa
aga    GUAAACA CCU   CUCUCAGCu      a
|||    ||||||| |||   |||||||||
ucu    CAUUUGU GGA   GAGGGUCga      g
   uUCU       C   --A         aagaau
```

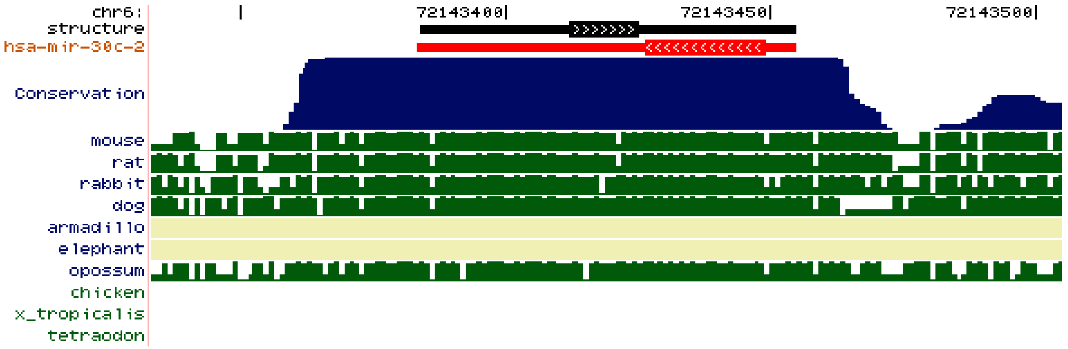

hsa-mir-628 chr15:53452430-53452524 (-)

```
auagcugu  u   a      cA   -   A   A             aaaa
        ug guc cuuccu  UG CUG CAU UUUACUAGAGGgu    u
```

```
        || ||| ||||||  || ||| ||| |||||||||||||
        ac cgg gaaggg  GC GAC GUG GAAUGAUCUucca    u
-------u  u   -      aA  U   G   A             auaa
```

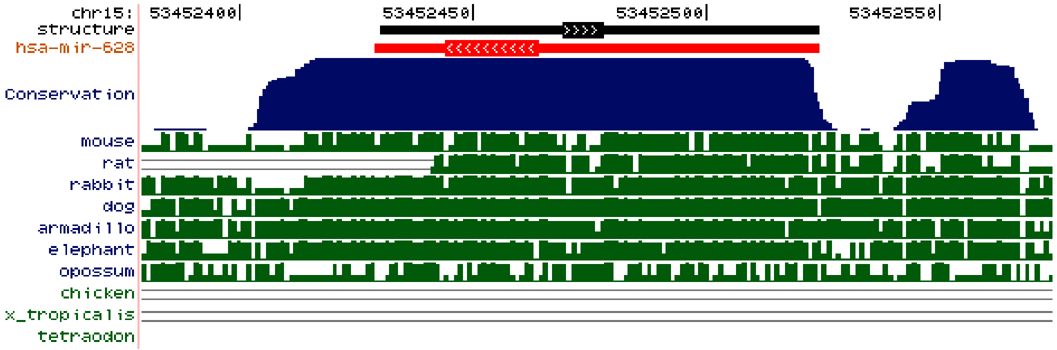

hsa-mir-18b chrX:133131737-133131807 (-)

```
ug     --   U    C   U     UA  --g
  uguUA  AGG GCAU UAG GCAGU  Gu   aagc
  |||||  ||| |||| ||| |||||  ||   ||| a
  aCGGU  UCC CGUA AUC CGUca  ua   uucg
--     CU   C    A   C     uc  aga
```

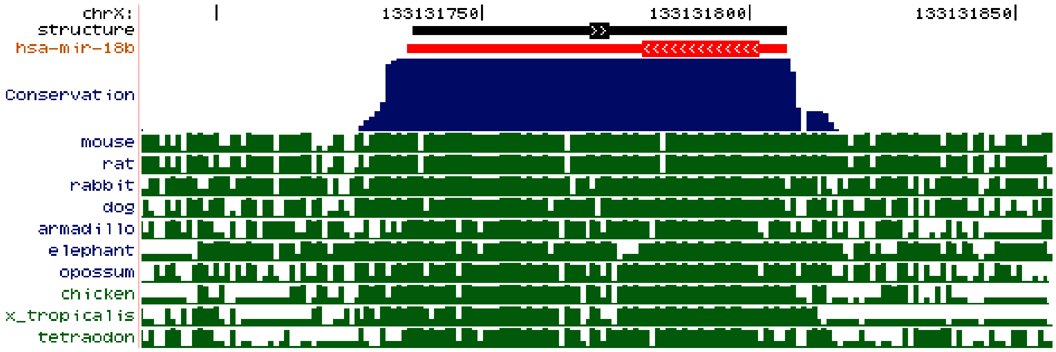

hsa-mir-10a chr17:44012199-44012308 (-)

```
-----gauc   --c    uu         A    G     C          uaaggaa
         ugu   uguc  cuguauaU CCCU UAGAU CGAAUUUGUG       u
         |||   ||||  |||||||| |||| ||||| ||||||||||
         aca   acag  gauguAUA GGGG AUCUA GCUUAAACac       u
ucucgccuc   aau    uu         A    -     U          ugguguu
```

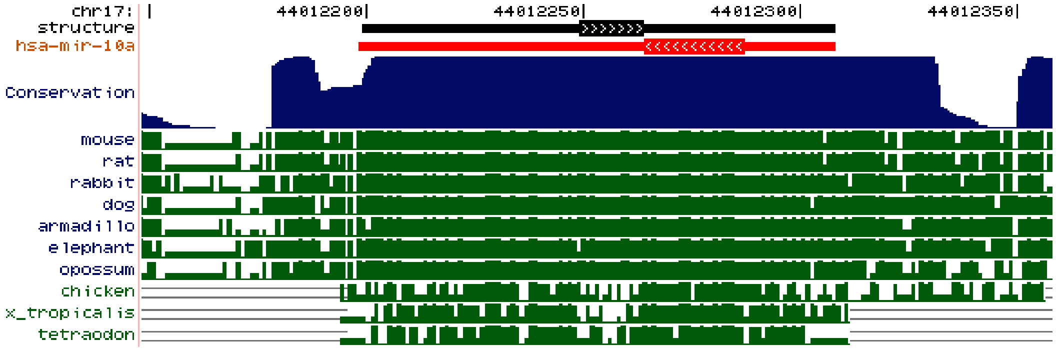

hsa-mir-30a chr6:72169975-72170045 (-)

```
   a            UC           -----   a
gcg cUGUAAACAUCC  GACUGGAAGcu     gug a
||| ||||||||||||  |||||||||||     |||
cgu GACGUUUGUAGG  CUGACUUUCgg     cac g
   C            --           guaga   c
```

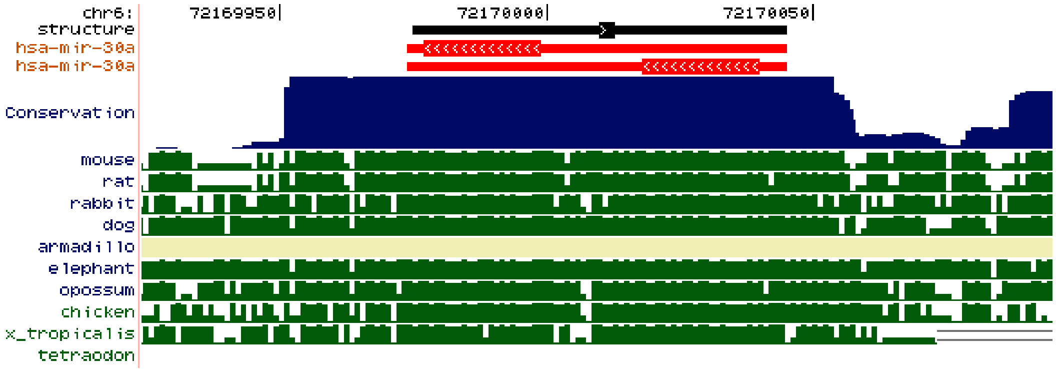

hsa-mir-874 chr5:137011160-137011237 (-)

```
--      cug   c   a  ca        a   -a a
  uuagcc   cgg ccc cg  ccagggua gag   g c
  ||||||   ||| ||| ||  |||||||| |||   | u
  ggucgg   GCC GGG GC  GGUCCCGU cuu   c c
cg      ucA   A   A  CC        C   cg u
```

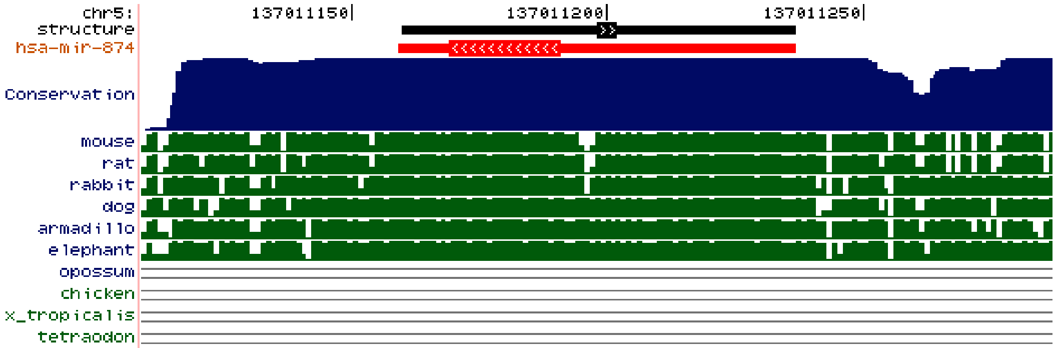

hsa-mir-147b   chr15:43512540-43512619 (+)

```
--  u    c   ug   a            aacuagauu
  ua aaau uag  gaa cauuucugcaca         cugga
  || |||| |||  ||| ||||||||||||         |||| c
  au uuua AUC  CUU GUAAAGGCGUGU         Gacca
gg  u    c   GU   C            ---------
```

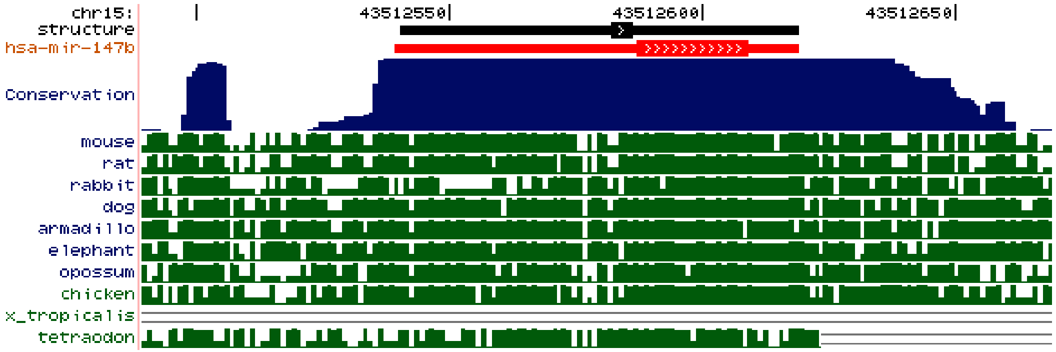

hsa-let-7c chr21:16834019-16834102 (+)

```
  a      uU   G   U             UA  G UA A
gc uccggg   GAG UAG AGGUUGUAUGGUU  GA U   C C
|| ||||||   ||| ||| |||||||||||||  || |   | C
cg agguuc   uuc auc uccaacaugucaA  UU A   G C
  -      cu   g   u             --  G GG U
```

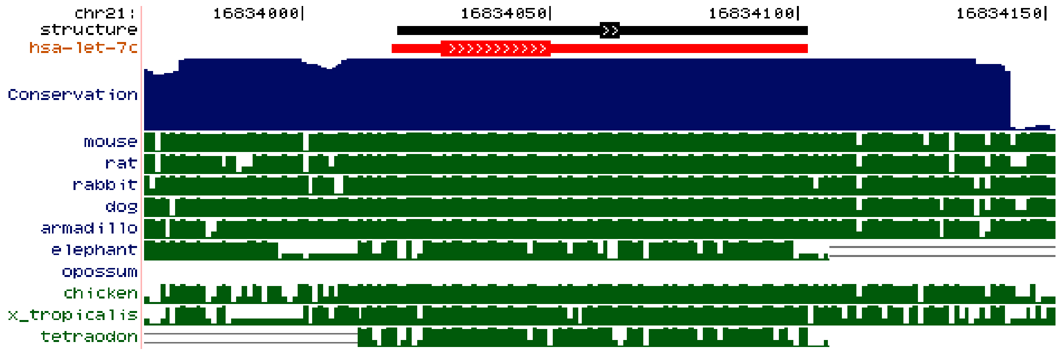

hsa-mir-136 chr14:100420792-100420873 (+)

```
u        g        C     UUU          uuc
 gagcccuc gaggACUC AUUUG   UGAUGAUGGA   u
 |||||||| |||||||| |||||   ||||||||||   u
 cuugggag cuUCUGAG UAAAC   GCUACUACcu   a
u        a        -     UCU          cgu
```

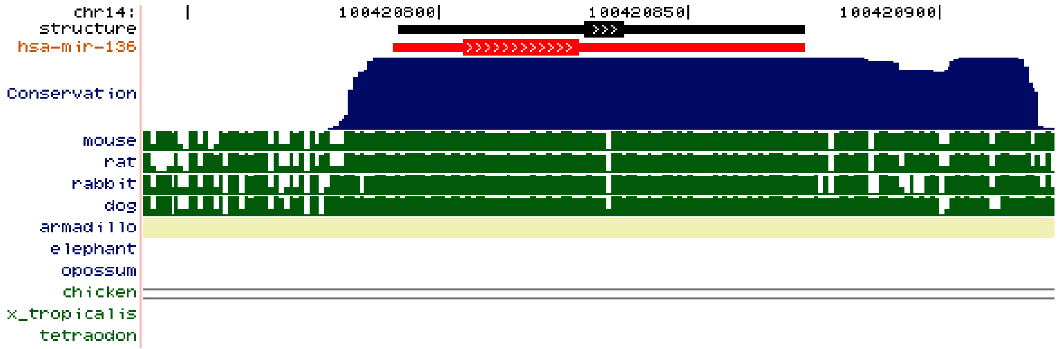

hsa-mir-361 chrX:85045297-85045368 (-)

```
     UU         --U  A    U     auaa
ggagc  AUCAGAAUC   CC GGGG ACuuu    u
|||||  |||||||||   || |||| |||||
cuucg  UAGUCUUAG   GG CCCC Ugaaa    u
     UU        UGU  A    C     aacu
```

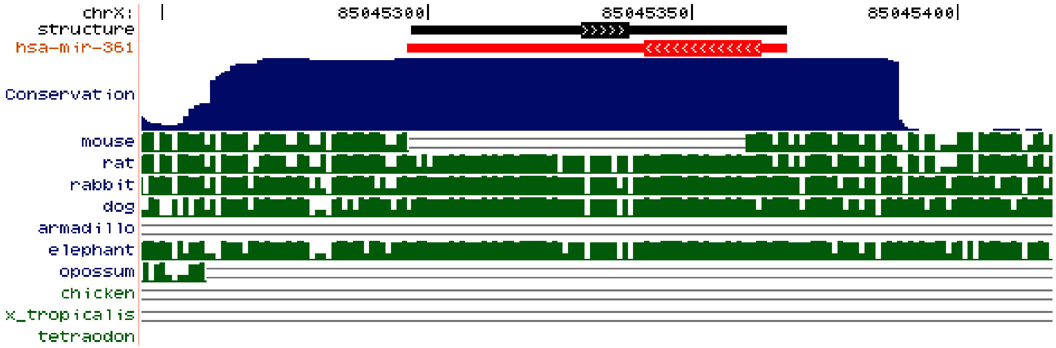

hsa-mir-101-2 chr9:4840297-4840375 (+)

```
  ug  c                        c a    guaua
ac  uc uuuuucgguuaucaugguac g ugcu     u
||  || ||||||||||||||||||| | ||||
ug  gg aagAAGUCAAUAGUGUCAUG C AUgg     c
  gu  u                        A -    aaagu
```

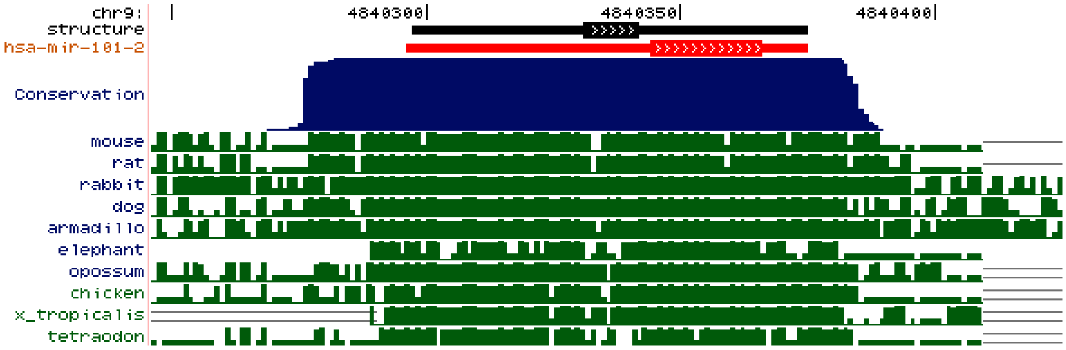

hsa-mir-15a chr13:49521256-49521338 (-)

```
     gaguaaa UA       UA          ga  u
ccuug       g  GCAGCACA  AUGGUUUGUG  uu u
|||||       |  ||||||||  ||||||||||  || g
ggaac       C  CGUCGUGU  UACCGGACgu  aa a
     auaaaaA UC       UA          gg  a
```

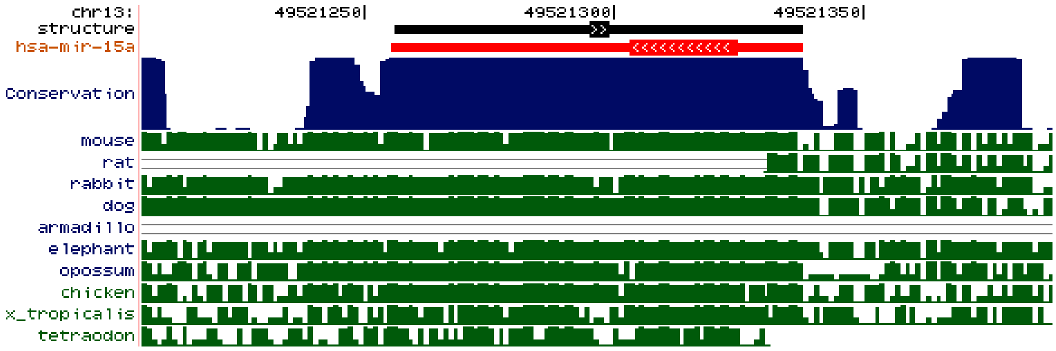

.

Figure S6. Multiple sequence alignments for the human genome with 16 other vertebrate genomes of human 74 pri-miRNAs that show high levels of conservation in their terminal loop regions.

.

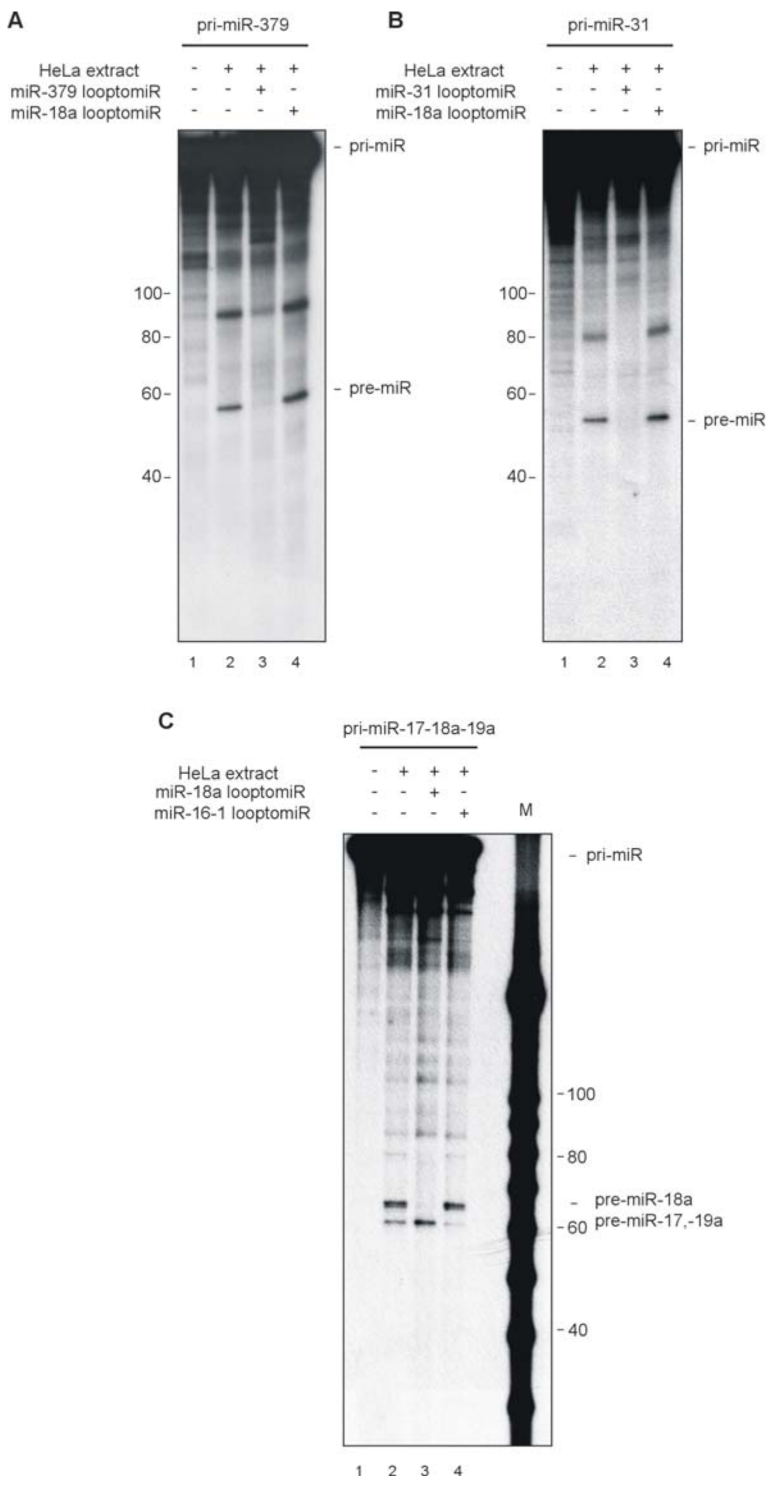

Figure S7. (A, B) The *in vitro* processing of pri-miRNAs-379 and pri-miR-31 is sensitive to the presence of specific looptomiRs. Radiolabeled pri-miR-379 and pri-

miR-31 ($50x10^3$ c.p.m.) were processed in HeLa extracts with the addition of a specific looptomiR (lanes 3) or with a control looptomiR specific for miR-18a (lanes 4). As a control, the reaction was also carried out in the absence of any looptomiR (lanes 2). Lanes 1 represent control reaction without extract. M, RNA size marker. (C) A miR-18a looptomiR selectively abolishes the processing of pri-miR-18a in the context of the miR-17-18a-19a mini cluster. The RNA substrate comprising pri-miR-17, pri-miR-18a and pri-miR-19a ($50x10^3$ c.p.m.) was processed *in vitro* in the presence of a miR-18a looptomiR (lane 3), or a control looptomiR (lane 4). Lane 1 shows negative controls with no extract added.

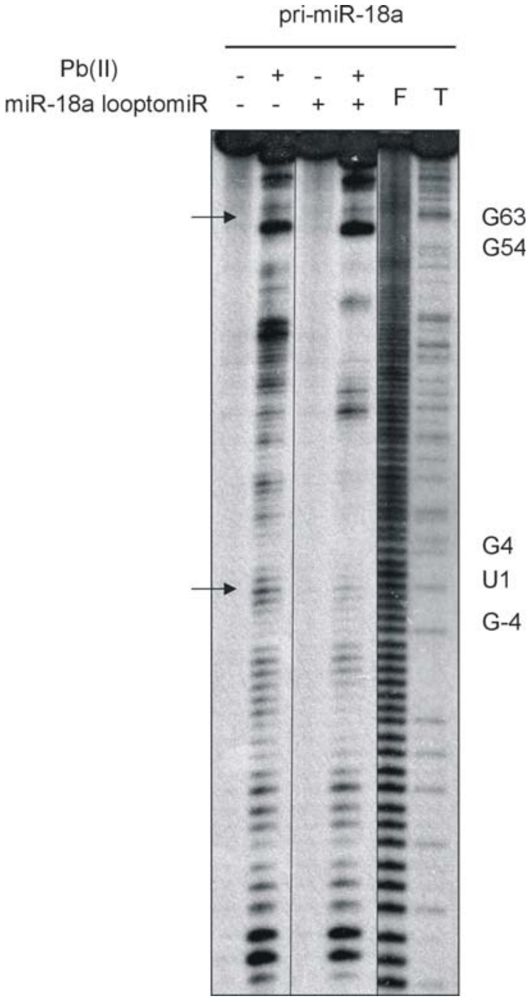

Figure S8. A LooptomiR targeting pri-miR-18a binds to the terminal loop region without disturbing RNA structure of the region surrounding the Drosha cleavage site. Cleavage patterns obtained for the 5’ ^{32}P-labeled pri-miR-18a transcript (100x10^3 c.p.m.) treated with Pb (II) – lead ions (0.5mM) with or without addition of specific looptomiR (50µM) are shown. Drosha cleavage sites are indicated with black arrows.

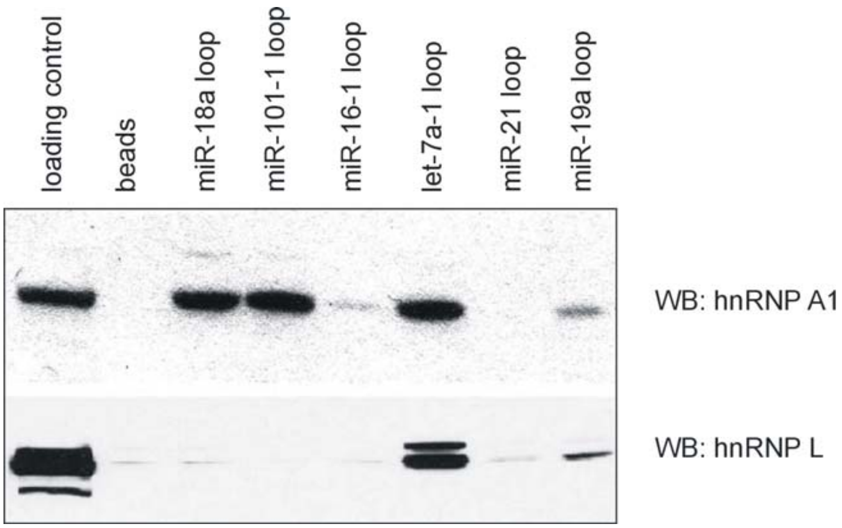

Figure S9. RNA chromatography of selected pri-miRNAs harboring conserved (miR-18a, miR-101-1 and let-7a-1) or not conserved (miR-16-1, miR-21, miR-19a) loops was performed in HeLa nuclear extracts. Samples resulting from RNA chromatography were resolved on SDS gel and visualized by Western blot analysis with specific antibodies against hnRNP A1 and hnRNP L.

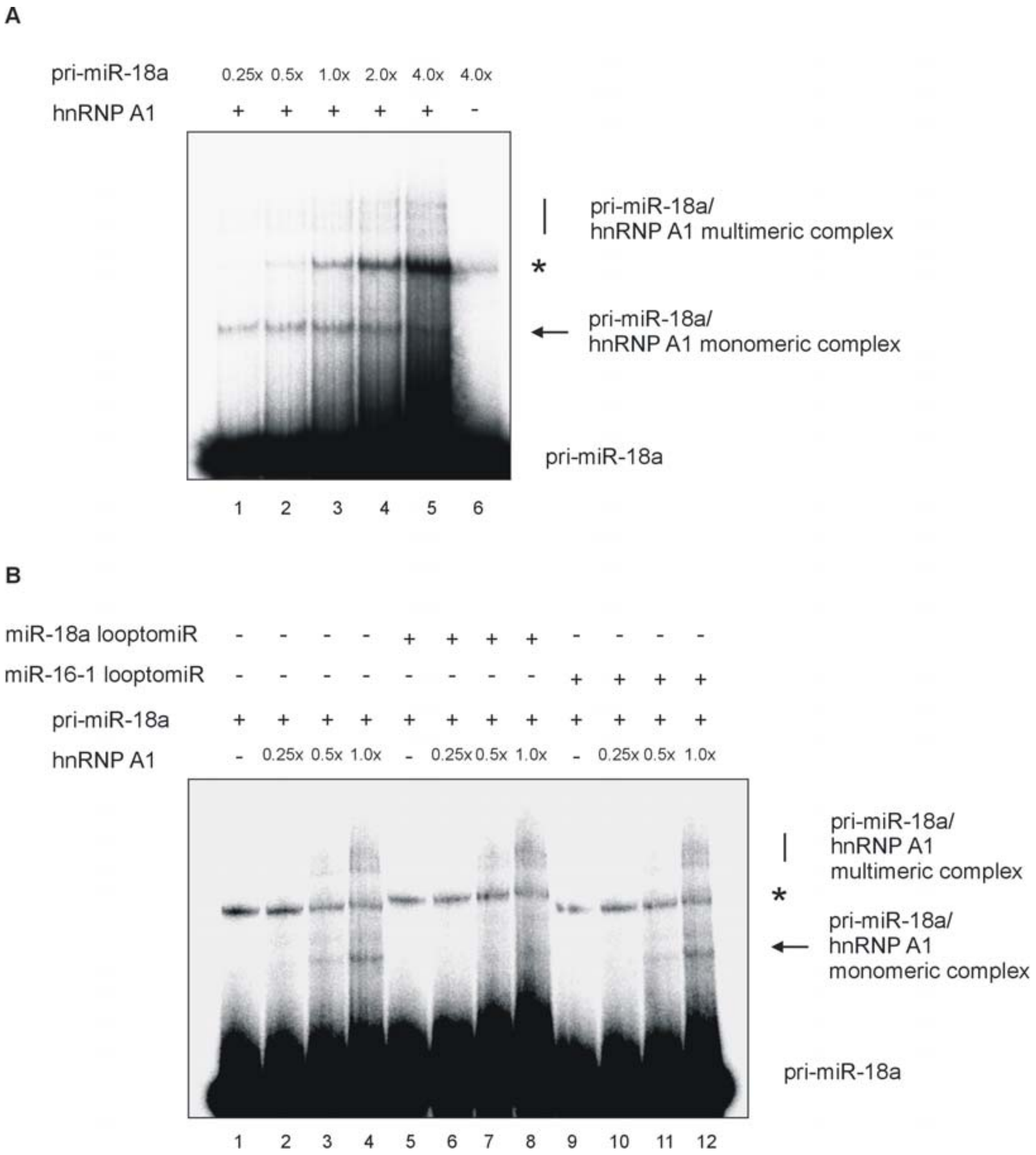

Figure S10. Several hnRNP A1 molecules bind pri-miR-18a and an specific looptomiR blocks the formation of one of the complexes. (A) EMSA analysis of pri-miR-18a transcript with recombinant hnRNP A1 protein. Native gel electrophoresis with increasing amounts of 5' ^{32}P-labeled transcript (25x10^3 c.p.m. up to 400x10^3 c.p.m./ 0.5 pmol up to 8.0 pmol) incubated in the presence of constant amounts of recombinant hnRNP A1 protein (the molar ratio indicated) reveals several, stoichiometrically different, complexes. The asterisk indicates the minor structural

conformer of pri-miR-18a transcript. (B). EMSA analysis of pri-miR-18a/hnRNP A1 complexes formed in the presence of looptomiRs. Equal amounts of 5' ^{32}P-labeled pir-miR-18a (100x10^3 c.p.m/ 2 pmol) incubated with increasing amounts of hnRNP A1 (0.5 pmol up to 2 pmol) were programmed with a specific LooptomiR targeting pri-miR-18a specific or acontrol looptomiR (50µM) and assayed as described above.

.

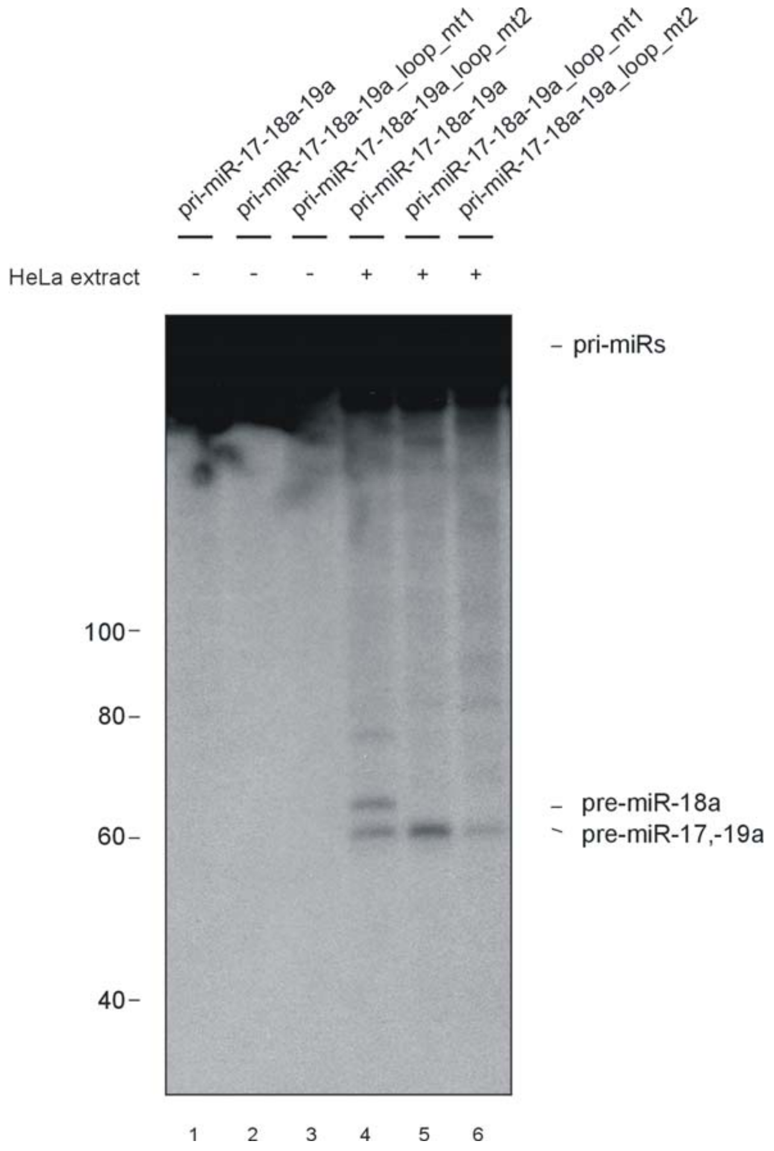

Figure S11. The conserved terminal loop of pri-miR-18a is required for its efficient cleavage by Drosha in the natural context of other pri-miRNAs. *In vitro* processing of pri-miR-17-18a-19a and pri-18a loop mutants in the mini cluster. Radiolabeled pri-RNAs ($50x10^3$ c.p.m.) were incubated in HeLa cell extracts (lanes 4, 5 and 6). Lanes 1, 2 and 3 show negative controls with no extract added. Products were analyzed on an 8% polycrylamide gel.

.

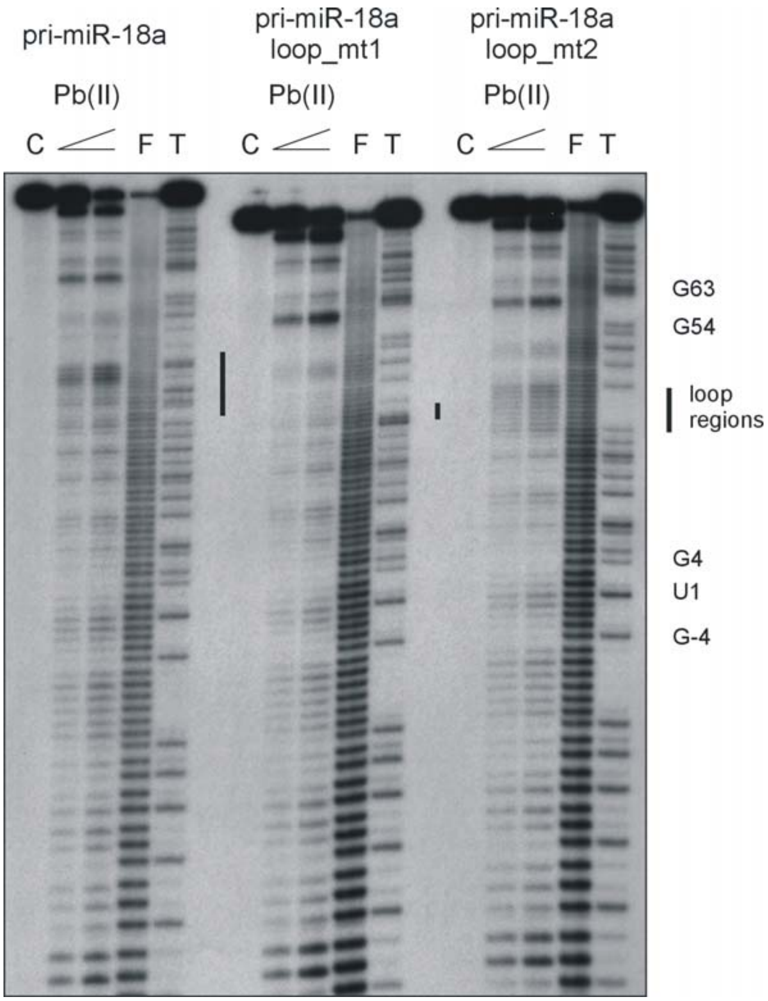

Figure S12. Structure analysis of wild-type and loop mutants of pri-miR-18a (A) Cleavage patterns obtained for the 5' ^{32}P-labeled pri-miR-18a (left panel) and pri-miR-18a loop mutant 1 (middle panel) and loop mutant 2 (right panel) transcripts ($100x10^3$ c.p.m.) treated with: Pb (II) – lead ions at increasing concentrations (0.5, 1 mM). C, incubation control (without probe). F and T identify nucleotide residues subjected to partial digest with formamide (every nucleotide) or ribonuclease T1 (G-specific cleavage), respectively. Electrophoresis was performed in a 10% polyacrylamide gel under denaturing conditions. The positions of selected G residues are shown. The loop regions are indicated by bars.

SUPPLEMENTAL EXPERIMENTAL PROCEDURES

Materials and Methods

Preparation of plasmids and DNA templates

For the structural analysis, the RNA substrates were transcribed *in vitro* from a DNA fragment containing the T7 promoter sequence directly upstream of the pri-miRNA precursor sequence. The DNA fragments were amplified by PCR from human genomic DNA using the following oligonucleotides: pri-miR-17: T7-1018for-G (5'-TTAATACGACTCACTATAGGCCATCACCTTGTAAAACTGAAG-3') and 80rev (5'- TAAAGCCCAACTTGGCTTCCCG-3'); pri-miR-19a: T7-236for-G (5'-TTAATACGACTCACTATAGGCGCCAAGCAAGTATATAGGTG-3') and 1446rev (5'- TTAGTAAAAATCATTCATTTG-3'); pri-miR-18a: T7-103for-G (5'-TTAATACGACTCACTATAGGCGTGCAGGGCCTGCTGATGTTGAGTGC-3') and 233rev (5'- GAATTATTGGATGAATACATAAC-3'); pri-miR-18b: T7-18bfor-G (5'- TTAATACGACTCACTATAGGCGCTTTTGAGCTGCTTCTTATAATG-3') and 18brev (5'- CTTTTAAAATGCTGTATATTAGGC-3'); pri-miR-18a_73nt: T7-1for-G (5'TTAATACGACTCACTATAGGTGTTCTAAGGTGCATCTAGT -3') and 71rev (5'- TGCCAGAAGGAGCACTTAG-3').

The sequences used for *in vitro* transcription and pri-miRNA processing assays were obtained by PCR from human genomic DNA, cloned in pGEM-T-easy (Promega) and linearized before transcription. The oligonucleotides used were as follows: pri-106a-18b-20b: clusterXfor (5'- CAGGAATATTAACTAGTAG-3') and clusterXrev (5'-ACGCTGAAATGCAAACCTGC-3'); pri-miR-17-18a-19a: 844for (5'-GAATTCTTAAGGCATAAATACG-3') and 1553rev (5'-GTAGATAACTAAACACTACC-3'); pri-miR-16_1: miR16for (5'-TGATAGCAATGTCAGCAGTGCC -3') and miR16rev (5'-

GTAGAGTATGGTCAACCTTAC-3’); pri-miR-27a: cl23-143for (5’-GGCAGAGAGGCCCCGAAGCC-3’) and cl23-279rev (5’-CAGGCGGCAAGGCCAGAGGAGG-3’); pri-let-7a-1: let-7a-1for (5’-GGAGCGGATTCAGATAACCA-3’) and let-7a-1rev (5’-CCAGGCCATAAACAAATGCT-3’); pri-miR-101-1: miR-101-1for (5’-GGGGAGCCTTCAGAGAGAGT-3’) and miR-101-1rev (5’-AGCCACCTGTTTCACATTCC-3’) pri-miR-31: miR-31for (5’-TCATCCCTGTGGTTTCACAA-3’) and miR-31revbis (5’-GGAAATCCACATCCAAGGAA-3’); pri-miR-379: miR-379for (5’-CAAATCCAGCCTCAGAAAGC-3’) and miR-379rev (5’-AGCCCAAGTTGCATCACTTC-3’). Mutants 18aUC>GU and 18bGU>CU were derived from the corresponding wild-type templates with oligonucleotides 18a_TC/GTfor (5’-TGAGTGCTTTTTGTGTTAAGGTGCATCTAG-3’) together with 18a_TC/GTrev (5’-CTAGATGCACCTTAACACAAAAAGCACTCA-3’) and 18b_GT/TCfor (5’-AATGTGTCTCTTGTTCTAAGGTGCATCTAG-3’) together with 18b_GT/TCrev (5’-CTAGATGCACCTTAGAACAAGAGACACATT-3’), respectively by site-directed mutagenesis (QuickChange, Stratagene). Templates for terminal loop mutants for pri-miR-18a, pri-miR-16-1, pri-miR-101-1 and pri-let-7a-1 were derived from the corresponding wild-type plasmids by PCR amplification with oligonucleotides 18a_loop_mt1for (5’-AATACTGCCCTAAGTGCTCCTT-3’) together with 18a_loop_mt1rev (5’-GCTATCTGCACTAGATGCACCT-3’); 18a_loop_mt2for (5’-CTCTACTGCCCTAAGTGCTCCTT-3’) together with 18a_loop_mt2rev (5’-AGATATCTGCACTAGATGCACCT-3’); 16_1_loop_mt1for (5’-AATTATCTCCAGTATTAACTGT-3’) together with 16_1_loop_mt1rev (5’-GCTTAACGCCAATATTTACGTG-3’); 16_1_loop_mt2for (5’-

CTCTTATCTCCAGTATTAACTGT-3') together with 16_1_loop_mt2rev (5'-AGATTAACGCCAATATTTACGTG-3'); 101-1_loop_mt1for (5'-AAGGTACAGTACTGTGATAACTGA -3') together with 101-1_loop_mt1rev (5'-GCAGCATCAGCACTGTGATAACTG -3'); 101-1_loop_mt2for (5'-CTCGGTACAGTACTGTGATAACTGA -3') together with 101-1_loop_mt2rev (5'-AGAAGCATCAGCACTGTGATAACTG -3'); let-7a-1_loop_mt1for (5'-AAAACTATACAATCTACTGTCTTTC -3') together with let-7a-1_loop_mt1rev (5'- GCAACTATACAACCTACTACCTCA -3'); let-7a-1_loop_mt2for (5'-CTCAACTATACAATCTACTGTCTTTC -3') together with let-7a-1_loop_mt2rev (5'- AGAAACTATACAACCTACTACCTCA -3'), using Pfu polymerase (Promega), followed by T4 ligation (Roche) and transformation of DH5alpha competent cells (Invitrogen).

RNA chromatography and western blotting.

Substrate RNAs let-7a-1 loop (5'-GUUGUAUAGUUUUAGGGUCACACCCACCACUGGGAGAUAACUAUACAAUC-3'); miR-101-1 loop (5'AGUGCUGAUGCUGUCUAUUCUAAAGGUACAGUACUG -3'); miR-379 loop (5'-AACGUAGGCGUUAUGAUUUCUGACCUAUGUA-3'), miR-21 loop (5'-GACUGAUGUUGACUGUUGAAUCUCAUGGCAACACCAGUCGA-3'); miR-19a loop (5'-UUGCACUACAAGAAGAAUGUAGUUGUGCAAAU-3'); miR-16-1 loop (5'-AAUAUUGGCGUUAAGAUUCUAAAAUUAUCUCCAGUAUUAA-3') purchased from Sigma-Aldrich were coupled to the agarose beeds (adipic acid dihidrasa - Sigma-Aldrich). Fisrt, 1 µmol of specific RNA was incubated for 1h in room

temperature in the mix conating 100 mM NaAc pH 5.0 and 5 mM of freshly prepared m-sodium periodate. Then, RNA was precipitated and resuspended in 100 mM NaAc pH 5.0. Meenwhile the agarose beeds were washed extensively and resusopened in enough volume of the 100 mM NaAc pH 5.0 to have a final mix of 50% beads. One voulme of agarose mix was added to two volumes of the m-periodate treated RNAs and left rocking over night at 4°C (All steps mentioned above were performed in eppendorf tubes wraped in alu foil to protect the oxidation of RNA from light). Non-bound RNA was washed once with the 2 M KCl, tiwce with the 1M KCl and three times with Roeder D (KCl 100mM, glicerol 20% p/v, EDTA 0,2mM, Hepes.KOH 100 mM pH 7,9, DTT 0,5 mM, protease inhibitors-Complete EDTA free (Roche)). To purify the factors from the nuclear extracts beads-coupled RNAs were incubated for 30min. at 37°C in the mixture containing 250µl (final 40% v/v) HeLa nuclear exctract (Cilbiotech), 1.5 mM $MgCl_2$, 0.5 mM ATP and 20 mM creatine phosphate. To analyze the factors bound to the RNA, -agarose beads were washed four times with 1ml Roeder D supplemented with 1.5 mM $MgCl_2$. Proteins were separated using NuPAGE Novex Bis-Tris system (Invitrogen). Protein bands visualized by GelCode Blue (Pierce) were subjected to Mass Spectroscopy (Dr Jim Creanor's service – Edinburgh University). Alternatively western blot analysis was carried out with hnRNP A1 – specific monoclonal antibody 4B10 (Abcam), with hnRNP L – specific monoclonal antibody 4D11 (Sigma-Aldrich) and hnRNP I/PTB – specific monoclonal antibody SH54 (Abcam).

EMSA analysis of pri-miR-18a/hnRNP A1 complexes. 5' ^{32}P-labeled pri-miR-18a transcripts (in the range from 20,000 c.p.m. to 400,000 c.p.m.) were incubated with recombinant hnRNP A1 (in the range from 50 ng to 200 ng) for 30 min in RT in the

buffer containing 0.5 mM ATP, 50 mM creatine phosphate, 5 mM $MgCl_2$ and 20 mg/ml heparin. When protein was not present the reactions were suplemented with corresponding volume of BC100. Where indicated 50µM looptomiR was added prior addition of the protein. The samples with added loading buffer (10mM Tris-HCl, 3% sucrose and dyes) were loaded on the 8% (w/v) non-denaturing gel and run in the constant power of 9W.
